# Deciphering of Adult Glioma Vulnerabilities through Expression Pattern Analysis of GABA, Glutamate and Calcium Neurotransmitter Genes

**DOI:** 10.3390/jpm12040633

**Published:** 2022-04-14

**Authors:** Hoang Dong Nguyen, Phedias Diamandis, Michelle S. Scott, Maxime Richer

**Affiliations:** 1 Département de Biochimie et de Génomique Fonctionnelle, Faculté de Médecine et des Sciences de la Santé, Université de Sherbrooke, Sherbrooke, QC J1E 4K8, Canada; hoang.dong.nguyen@usherbrooke.ca (H.D.N.); michelle.scott@usherbrooke.ca (M.S.S.); 2 Department of Laboratory Medicine and Pathobiology, University of Toronto, Toronto, ON M5S 1A8, Canada; p.diamandis@mail.utoronto.ca; 3 Princess Margaret Cancer Center, University Health Network, Toronto, ON M5G 2C1, Canada; 4 Institute of Medical Science, University of Toronto, Toronto, ON M5S 1A8, Canada; 5 Axe Neurosciences du Centre de Recherche du Centre Hospitalier Universitaire (CHU) de Québec—Université Laval et Département de Biologie Moléculaire, Biochimie et Pathologie de l’Université Laval, Québec, QC G1V 4G2, Canada

**Keywords:** glioma, tumor immune microenvironment, transcriptomics, methylation, amino acid neurotransmission, GABA, glutamate, calcium

## Abstract

Adult infiltrating gliomas are highly aggressive tumors of the central nervous system with a dismal prognosis despite intensive multimodal therapy (chemotherapy and/or radiotherapy). In this study, we studied the expression, methylation and interacting miRNA profiles of GABA-, glutamate- and calcium-related genes in 661 adult infiltrating gliomas available through the TCGA database. Neurotransmitter-based unsupervised clustering identified three established glioma molecular subgroups that parallel major World Health Organization glioma subclasses (IDH-wildtype astrocytomas, IDH-mutant astrocytomas, IDH-mutant oligodendroglioma). In addition, this analysis also defined a novel, neurotransmitter-related glioma subgroup (NT-1), mostly comprised of IDH-mutated gliomas and characterized by the overexpression of neurotransmitter-related genes. Lower expression of neurotransmission-related genes was correlated with increased aggressivity in hypomethylated IDH-wildtype tumors. There were also significant differences in the composition of the tumor inflammatory microenvironment between neurotransmission-based tumor categories, with lower estimated pools of M2-phenotype macrophages in NT-1 gliomas. This multi-omics analysis of the neurotransmission expression landscape of TCGA gliomas—which highlights the existence of neurotransmission-based glioma categories with different expression, epigenetic and inflammatory profiles—supports the existence of operational neurotransmitter signaling pathways in adult gliomas. These findings could shed new light on potential vulnerabilities to exploit in future glioma-targeting drug therapies.

## 1. Introduction

Infiltrating gliomas are the most common malignant tumors of the central nervous system in adults. They represent nearly 45–50% of malignant primary brain neoplasms [1] and are associated with relatively short survivals [2]. The infiltrative nature, intratumoral heterogeneity [3] and cellular signaling complexities of these aggressive tumors make them a major challenge to overcome in terms of therapy and personalized patient management [4,5]. Pathological and adaptative interactions with the surrounding microenvironment (non-neoplastic glia, neurons and immune cells) further contribute to tumor aggressivity and progression [6].

Current World Health Organization diagnostic classification schemes for adult infiltrating gliomas are based on the presence of molecular alterations such as isocitrate dehydrogenase 1/2 mutations (IDH1/2) and 1p/19q codeletion [1]. While IDH-wildtype gliomas usually present with high-grade histology and correlate to short survival [7,8], IDH-mutated, 1p/19q-non-codeleted astrocytomas and IDH-mutated 1p/19q-codeleted oligodendrogliomas are usually first diagnosed as low-grade gliomas and associated with longer survival [7,9].

IDH1/2 genes code for isocitrate dehydrogenase cytoplasmic enzymes that play a crucial role in cellular energy metabolism^7^. One of their major functions is the catalysis of citrate oxidative decarboxylation to alpha-ketoglutarate (α-KG), which is a well-known intermediate of the TCA cycle [10]. Further, α-KG is involved in neurotransmission by serving as a precursor of two important human brain neurotransmitters: glutamate and gamma-aminobutyric acid (GABA) [11]. In glioma, mutations in IDH1/2 lead neoconversion of α-KG into the oncometabolite *D*-2-hydroxyglutarate (*D*-2-HG) [12]. Consequently, the IDH status of gliomas impacts not only their energetic metabolism but also their integration into surrounding neural circuits due to the potential dysregulation in neurotransmitter metabolism, thereby affecting tumor progression [13].

Aside from their leading role in neuronal synaptic transmission, GABA and glutamate can regulate many other brain biological processes. Several lines of evidence point to an important modulatory role of GABA and glutamate systems on neuroinflammatory processes [14] in the mature brain. This connection to signaling mechanisms of immunity makes them important therapeutic targets to reverse the detrimental effects of chronic neuroinflammation, as it is pivotal to the pathophysiology of numerous diseases of the central nervous system. In the developing brain, both molecules modulate neural precursor cell proliferation, differentiation and neuron migration through a reciprocal relationship with neurotransmitter-sensitive immune cells such as microglia [15,16]. Signaling mechanisms involved in these processes are thought to be operational in glioma [17].

Starting with the postulate that neurotransmission signaling activity is of importance to glioma biology, we initially interrogated the transcriptome of 661 gliomas available through the TCGA database for GABA-, glutamate- and calcium-related gene expression patterns. Four glioma clusters were generated by unsupervised clustering and compared to established glioma molecular diagnostic subgroups, as per the World Health Organization glioma classification. We subsequently used theses analyses as a starting point in deciphering ties to epigenetic (DNA methylation and microRNA) and immune mechanisms to find novel vulnerabilities.

## 2. Materials and Methods

### 2.1. Sample Extraction

Data from the low-grade glioma [18] (LGG) and glioblastoma multiforme [19] (GBM) projects were extracted from the TCGA (The Cancer Genome Atlas, https://portal.gdc.cancer.gov/, accessed on 1 September 2020). These datasets include clinical and epidemiological data, gene and miRNA expression, methylation quantification, copy number variation and simple nucleotide variation. Biomolecular information (TERT promoter, ATRX, MGMT promoter status) extracted from the Ceccarelli study [20] was also added to our analysis.

Two RNA-seq datasets were extracted from the Chinese Glioma Genome Atlas [21,22,23] (CGGA, http://cgga.org.cn/, accessed on 1 September 2020) and were merged into one dataset to simplify the analysis. Clinical data (sex, age or overall survival) associated with these samples were also extracted.

Glioma samples from both databases (TCGA and CGGA) were annotated according to the IDH mutation and 1p/19q codeletion status: IDH-mutated with the 1p/19q co-deletion gliomas (IDH-MUT codel), IDH-mutated without the 1p/19q codeletion (IDH-MUT non-codel) and IDH-wildtype (IDH-WT). The TCGA dataset was used for gene expression, miRNA expression and DNA methylation profiling. CGGA was used for validation purposes.

### 2.2. GABA, Glutamate and Calcium Pathway Gene Extraction

The KEGG pathway database was the source to identify genes associated with GABA, glutamate and calcium metabolic pathways [24] (https://www.genome.jp/kegg/pathway.html, accessed on 1 September 2020). The following KEGG identifiers were used for extraction: 04727 (GABAergic synapse pathway); 04724 (glutamatergic synapse); 00250 (alanine, aspartate and glutamate metabolism), 04020 (calcium signaling) and 04961 (other factor-regulated calcium reabsorption pathways).

### 2.3. Gene Expression Normalization

Expression normalization was performed to enable comparisons between glioma samples. TCGA raw-count expression data were normalized using the Variance Stabilizing Transformation function available in the DESeq2 [25] v1.26.0 R package. Low-expression genes whose maximum did not pass 10 counts were excluded.

### 2.4. Unsupervised Clustering

Unsupervised clustering was performed to reveal hidden patterns of neurotransmission-related gene expression. Entropy values were calculated, varying the cluster number in order to select the optimal number of clusters that minimized the heterogeneity of IDH mutation and 1p19q codeletion within a cluster. Both unsupervised clustering and heatmaps were generated using the Complex Heatmap [26] v2.4.2 R package, based on the Ward method and Spearman correlation as distance.

### 2.5. Differential Gene Expression Analysis

The DESeq2 [25] R package v1.26.0 was used to retrieve differentially expressed genes between clusters. Bonferroni-corrected *p*-values below 0.05 were considered significant. Volcano plots of differential gene expression were generated using the EnhancedVolcano v1.8.0 R package.

### 2.6. miRNAs Interactome Profiling

MiRNAs interacting with the neurotransmission-related gene set were extracted from the RNA Interactome database [27]. Using the TCGA miRNA dataset, differential gene expression analysis was performed to filter differentially expressed miRNAs between identified glioma clusters (DESeq2 Bonferroni-adjusted *p* < 0.001 and absolute log2 fold change superior to 1). MiRNAs with an average expression lower than 1 RPKM were not included in the analysis.

### 2.7. Statistical Analysis

Statistical analyses were performed using R v4.0.3. Ggplot2 v3.3.5 and UpsetR v1.4.0 R packages were used for figure generation. We executed survival and Cox regression analyses using “survminer” v0.4.8 and “survival” v3.2.7 R packages.

### 2.8. Snakemake Pipepeline Creation

The study pipeline was built using snakemake [28] workflow manager v5.32.0. The software and packages used in the pipeline were downloaded from the bioconda channel via the package manager conda, and steps with high computational cost were executed using Compute Canada structures. This pipeline is available in GitHub at this address: https://github.com/hoang31/gaba_glutamatate_TCGA_profiling.git (accessed on 1 September 2020).

## 3. Results

### 3.1. Unsupervised Clustering Based on GABA, Glutamate and Calcium Gene Expression Distinguishes Four Clusters with Distinct Neurotransmission Profiles

**Figure 1:**
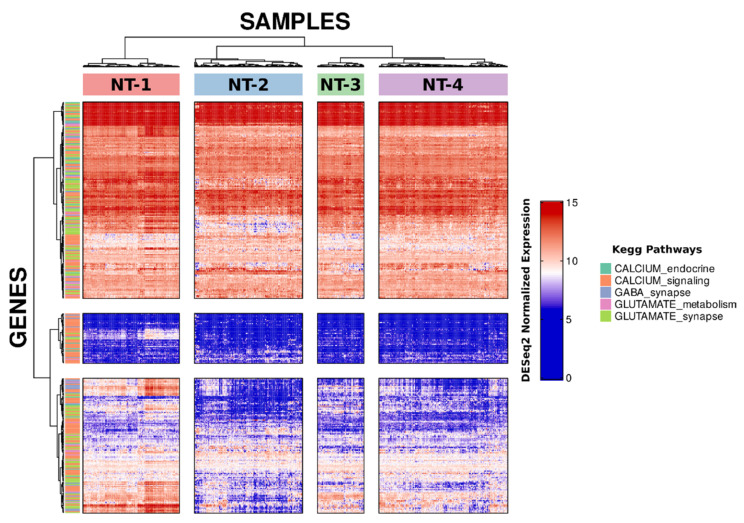
GABA, glutamate and calcium pathway-related gene expression signatures of 661 TCGA gliomas samples. Four clusters were generated by hierarchical unsupervised clustering using Pearson correlation (glioma samples in column) and Minkowski distance (genes in rows). Ward.D2 clustering method was selected for this clustering. Colors in rows represent different KEGG metabolic pathways of the included neurotransmission genes.

A total of 421 neurotransmission-related genes were extracted from the KEGG metabolic database, more specifically from four metabolic pathways: GABAergic neuron (kegg id: 04727), glutamatergic neuron (kegg id: 04724), glutamate metabolism (kegg id: 00250) and calcium signaling and endocrine (kegg ids: 04020 and 04961) pathways. Of these, 351 minimally expressed genes were kept (Supplementary Data Table S1), and there was limited overlap between the different gene sets (Supplementary Data Figure S1). Unsupervised clustering of 661 TCGA glioma samples was then performed in order to evaluate the expression pattern of these genes (Figure 1). IDH and 1p/19q codeletion-based entropy analysis showed an optimal number of clusters equal to four (Supplementary Data Figure S2). We thus generated four clusters, which were renamed NT-1 (*n* = 168), NT-2 (*n* = 188), NT-3 (*n* = 81) and NT-4 (*n* = 224) for neurotransmission-related clusters (Supplementary Data Table S2). This clustering analysis identified four main clusters that differ greatly in their neurotransmission-related gene expression profiles.

### 3.2. Neurotransmission-Based Glioma Clustering Recapitulates Current Existing Glioma Molecular Subgroups and Identifies a Novel Subgroup with a Distinct Expression Profile

**Figure 2:**
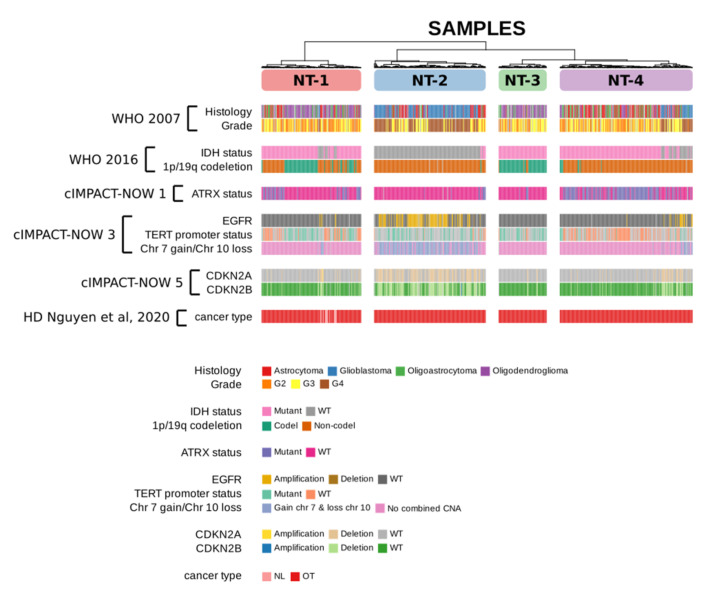
Distribution of frequent glioma molecular alterations within the four subgroups identified by GABA, glutamate and calcium gene expression-based unsupervised clustering. The molecular information for NT-1-4 gliomas was in accordance with the latest recommendations of the WHO classification of tumors of the central nervous system.

We evaluated the presence of the most frequent adult glioma molecular alterations in neurotransmission-based glioma subgroups in accordance with the latest WHO classification of tumors of the central nervous system [1]. We also tracked “normal-like” IDH-wt gliomas that had been identified in our previous study and were associated with a longer survival [29] (Figure 2 and Supplementary Data Table S3).

The different histopathological entities (as per the 2007 WHO classification of CNS tumors [30], i.e., oligodendroglioma, glioblastoma, astrocytoma and oligoastrocytoma) were heterogeneously distributed in the four NT clusters, with NT-2 gliomas being mainly comprised of glioblastomas (115/188 samples, 61.2%). NT-2, NT-3 and NT-4 gliomas were essentially composed of IDH-wt gliomas (180/188 samples, 95.7%), IDH-mutated gliomas with the 1p/19q co-deletion (75/81 samples, 92.6%) and IDH-mutated gliomas without the 1p/19q co-deletion (176/224 samples, 78.6%), respectively. As for the NT-1 glioma cluster, it was composed of a majority of IDH-mutated gliomas: 80/168 samples, 47.6% for IDH-mutated gliomas with the 1p/19q co-deletion; 67/168 samples, 39.9% for IDH-mutated gliomas without the 1p/19q co-deletion; and 21/168 samples, 12.5% for IDH-wt gliomas. The percentage of IDH-wt gliomas in NT-1 and NT-4 was similar (12.5% and 16.5%, respectively). The ATRX mutation was principally found in NT-4 (133/224 samples, 59.4%) and NT-1 glioma clusters (46/168 samples, 27.4%), as expected. According to available data, EGFR amplifications and combined Chr7 gain/Chr10 loss were essentially present in NT-2 gliomas (89/188 samples, 47.3% and 129/188 samples, 68.6%, respectively). The TERT promoter mutation was more prevalent in NT-2 (54/188, 28.7% versus 10/188, 5.3% for wildtype) and NT-3 clusters (32/81, 39.5% versus 5/81, 6.2%). NT-2 gliomas were also enriched with CDKN2A/B-deleted gliomas (105/188 samples, 55.9% and 103/188 samples, 54.8%, respectively). Interestingly, all the identified IDH-wt “normal-like” gliomas [29] were ascribed to the NT-1 glioma cluster. When specifically examining IDH-wt gliomas ascribed to NT-1-4 clusters, we found a higher proportion of IDH-wt gliomas bearing an EGFR amplification observed when comparing NT-2 vs. NT-1 gliomas but not NT-4 (49.44%, 23.81% and 40.54% for NT-1, NT-2 and NT-4, respectively; *p* = 1.88 × 10^−2^ and *p* = 0.25). Chr 7 gain/Chr 10 loss was also less prevalent in IDH-wt gliomas ascribed to the NT-1 cluster when compared to NT-2 (28.57% for NT-1 vs 71.66% and 45.94% for NT-2 and NT-4, respectively; *p* = 3.15 × 10^−4^ and *p* = 0.36). We conclude that the expression levels of GABA, glutamate and calcium signaling elements segregate with known glioma molecular alterations (IDH mutation and 1p/19q chromosomic status) for NT-1, NT-2, NT-3 and NT-4 clusters.

### 3.3. Clinical and Epidemiological Characterization of Neurotransmission-Based Glioma Clusters

We evaluated the clinical and histopathological characteristics of the four glioma subgroups identified based on their expression patterns of glutamate, GABA and calcium genes. Comparison analysis (Table 1) did not show significant age differences between the NT-1, NT-3 and NT-4 gliomas (average of 42.69 yo, *p* = 0.08 and 0.18). On the other hand, NT-2 gliomas affected significantly older patients (average of 58.12 yo, *p* = 1.06 × 10^−21^; *p* = 4.29 × 10^−12^; *p* = 9.95 × 10^−29^). Gliomas were more frequent in males than females, with no significant gender distribution differences between the different clusters.

**Figure 3:**
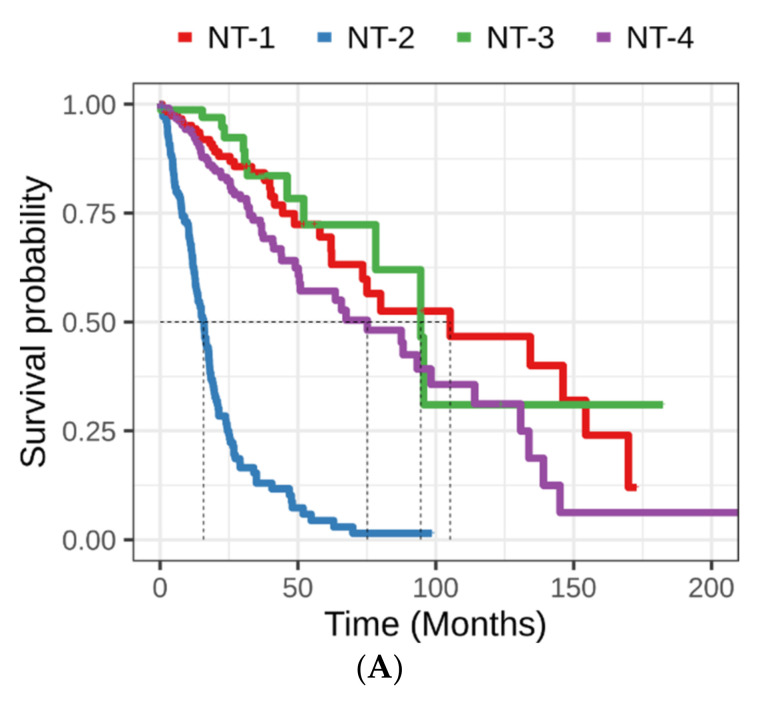

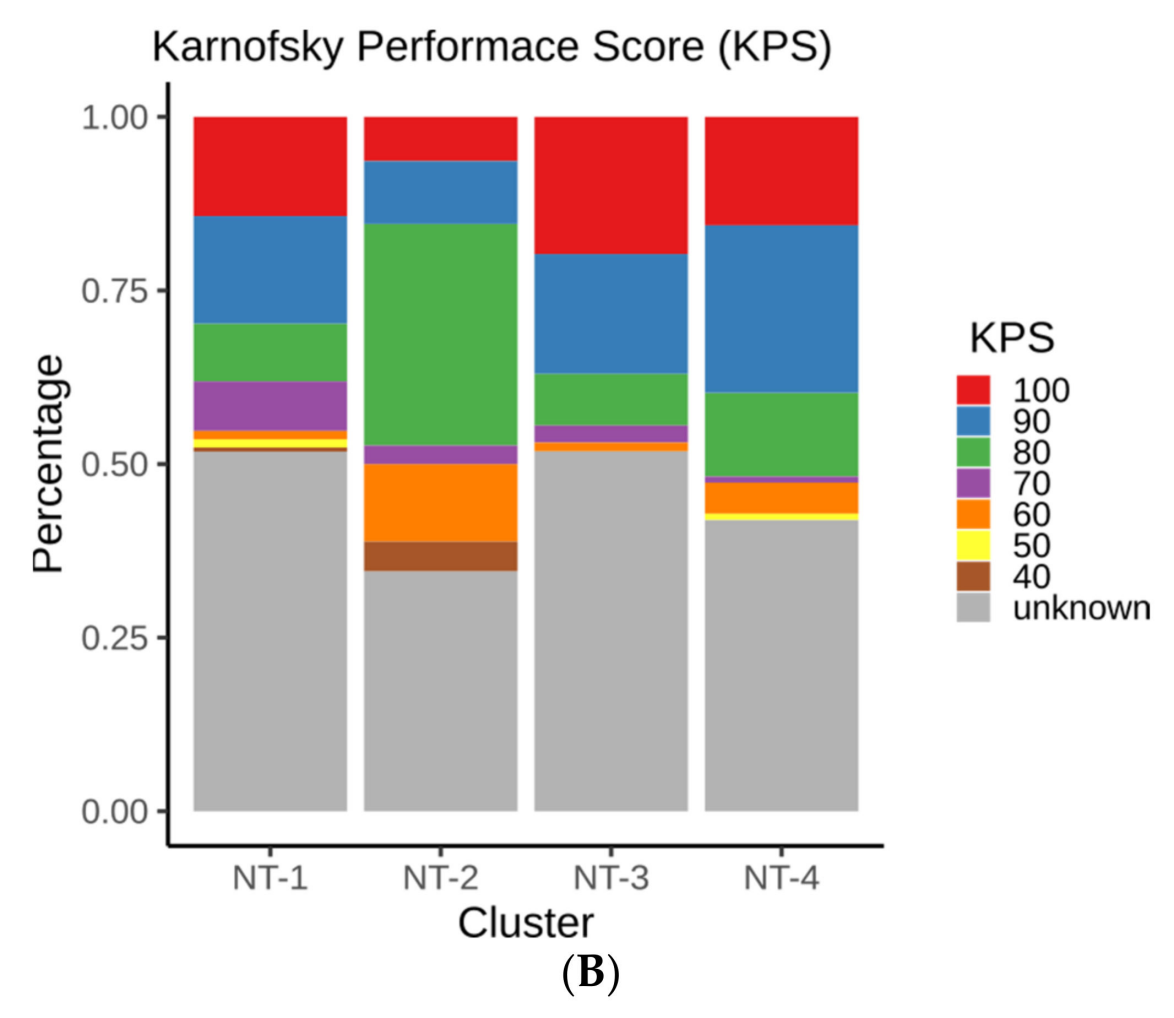
(**A**) Kaplan–Meier survival curves associated with NT-1-4 gliomas. Red, blue, green and purple represent the various NT-1-4 glioma clusters, respectively. Cluster-to-cluster significance was calculated using the log-rank test. (**B**) Karnofsky performance score distribution among NT-1-4 gliomas. These scores reflect the patient’s ability to perform ordinary tasks and range from 100 (patient without disabilities) to 0 (patient death).

Survival analysis showed similar survival for NT-1 and NT-3 (log-rank test *p* = 0.44, Figure 3A). However, survival was shorter for NT-2 gliomas (log-rank test, *p* = 7.10 × 10^−32^, *p* = 1.06 × 10^−20^ and 1.09 × 10^−33^ compared to NT-1, NT-3 and NT-4, respectively). The same pattern was observed with regard to the Karnofsky’s performance scores associated with the different glioma NT clusters; NT-2 glioma patients were associated with significantly lower performance scores (Fisher’s exact test, *p* = 5.00 × 10^−4^ Figure 3B).

We performed a univariate and multivariate cox regression analysis to validate the prognosis associated with each glioma cluster (Table 2). As expected from the literature, univariate regression suggested that higher age at diagnosis, clustering within the NT-2 cluster or higher grade had significant negative impact on patient survival (beta = 0.066 with *p* = 9.31 × 10^−42^; beta = 2.216 with *p* = 6.62 × 10^−28^; beta = 2.976 with *p* = 6.38 × 10^−43^ for NT-2 cluster, G4 glioma and age at diagnosis, respectively). The multivariate cox regression model confirmed that clustering within the NT-2 glioma cluster is an independent factor impacting patient survival (beta = 0.901; *p* = 1.93 × 10^−4^) regardless of gender, age or grade. As expected, age at diagnosis and grade were also found to be independent, significant prognostic factors for gliomas. NT-2 gliomas, which are mostly comprised of IDH-wt gliomas, bear the worst prognosis out of the neurotransmission-related clusters. NT-1 gliomas had similar prognosis compared to NT-3 and NT-4 gliomas.

### 3.4. Lower Expression of Neurotransmission Genes Correlates with Increased Aggressivity in the NT-1, NT-2, NT-3 and NT-4 Gliomas

**Figure 4:**
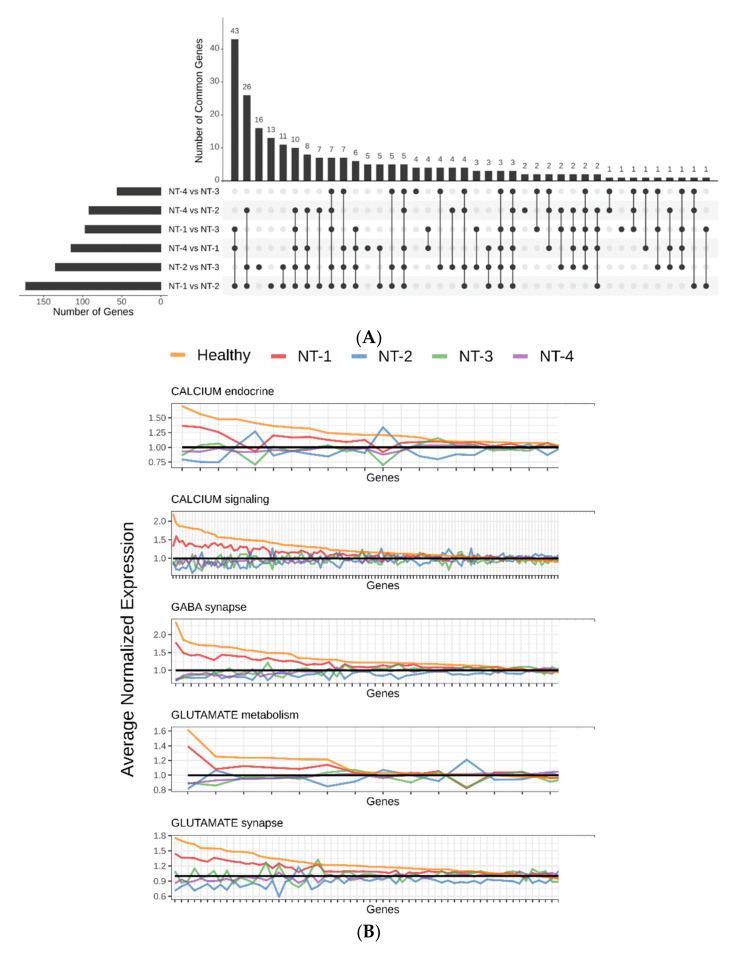

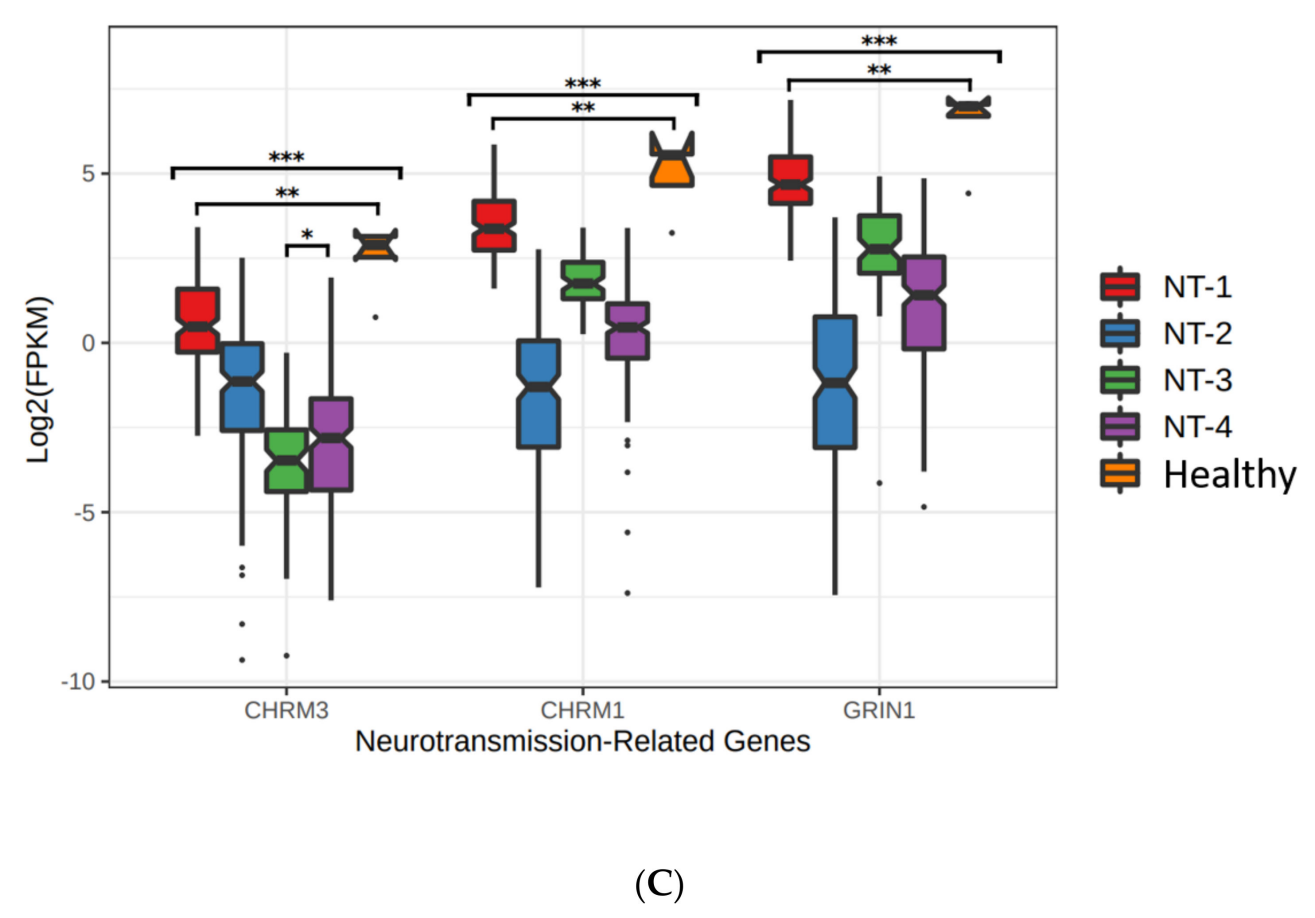
(**A**) Upset plot representing the differential gene cluster-to-cluster expression analysis. A total of 232 genes were differentially expressed in NT-1-4 gliomas (up- or downregulated). Each row represents a specific cluster comparison; columns represent the number of common genes. Each linked black point represents intersecting genes between glioma clusters. (**B**) GABA-, glutamate- and calcium-related gene expression profiles for NT-1-4 gliomas. Average normalized expression was performed with the DESeq2 v1.26.0 R package. Healthy samples (*n* = 5) were also included in this analysis. Genes were ordered by descending expression levels of the healthy samples. (**C**) CHRM1, CHRM3 and GRIN1 gene expression levels in NT-1-4 gliomas. Expression levels are presented in log2 FPKM for 3 intersecting genes from all 6 cluster-to-cluster expression analyses (* *p* < 0.05, ** *p* < 0.001 and *** *p* < 0.0001).

Following the identification of four glioma clusters with distinct GABA, glutamate and calcium-related gene expression patterns, we searched for specific genes expressed by cancer cells or their microenvironment that may impact gliomagenesis by performing differential gene cluster-to-cluster expression analysis. We retrieved 232 unique neurotransmission-associated genes that were significantly expressed (Bonferroni-adjusted *p* < 0.001 and absolute value of log2foldchange greater than 1). In this analysis, the highest number of significantly differentially expressed genes (43 genes) belonged to the NT-1 cluster comparison (Figure 4A).

Cluster-to-cluster expression profile analyses for calcium endocrine, calcium signaling, GABA synapse, glutamate metabolism and glutamate synapse metabolic pathways are presented in Figure 4B and Supplementary Data Table S4. Amongst all samples (glioma and healthy patients), we observed that neurotransmission-related genes were the most highly expressed in healthy samples when compared to NT glioma clusters. (18/23, 82/132, 43/55, 6/15 and 49/65 genes for calcium endocrine, calcium signaling, GABA synapse, glutamate metabolism and glutamate synapse metabolic pathways, respectively). With regard to the comparisons between NT glioma clusters, we found that NT-1 gliomas were associated with the largest number of overexpressed genes (15/23, 70/132, 41/55, 8/15 and 43/65 genes for calcium endocrine, calcium signaling, GABA synapse, glutamate metabolism and glutamate synapse metabolic pathways, respectively). Conversely, NT-2 gliomas were associated with the largest number of underexpressed genes ascribed to these pathways (14/23, 60/132, 36/55, 7/15 and 48/65 for calcium endocrine, calcium signaling, GABA synapse, glutamate metabolism and glutamate synapse metabolic pathways, respectively). These observations were more significant for GABA synapse, glutamate metabolism and glutamate synapse pathways. NT-3 and NT-4 cluster expression profiles showed intermediate levels of expression for all pathways. We also identified three genes that were significantly differentially expressed amongst all six cluster-to-cluster analyses: the cholinergic receptor muscarinic 1 (CHRM1), the cholinergic receptor muscarinic 3 (CHRM3) and the glutamate ionotropic receptor NMDA type subunit 1 (GRIN1) genes. These three genes were significantly overexpressed in healthy samples and NT-1 gliomas (Figure 4C). Overall, we found that NT-1 gliomas are characterized by the overexpression of neurotransmission-related genes when compared to other glioma clusters.

### 3.5. Correlation between DNA Hypermethylation and Gene Expression Is Preserved in NT-1 Gliomas

**Figure 5:**
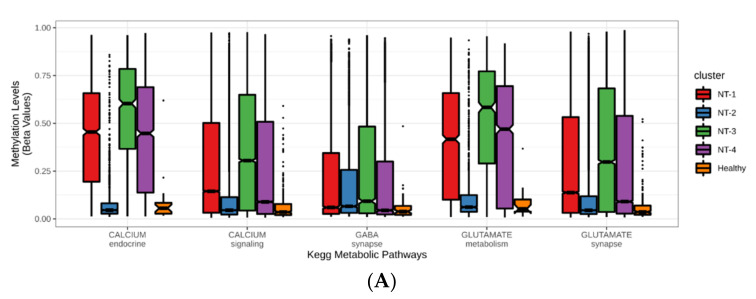

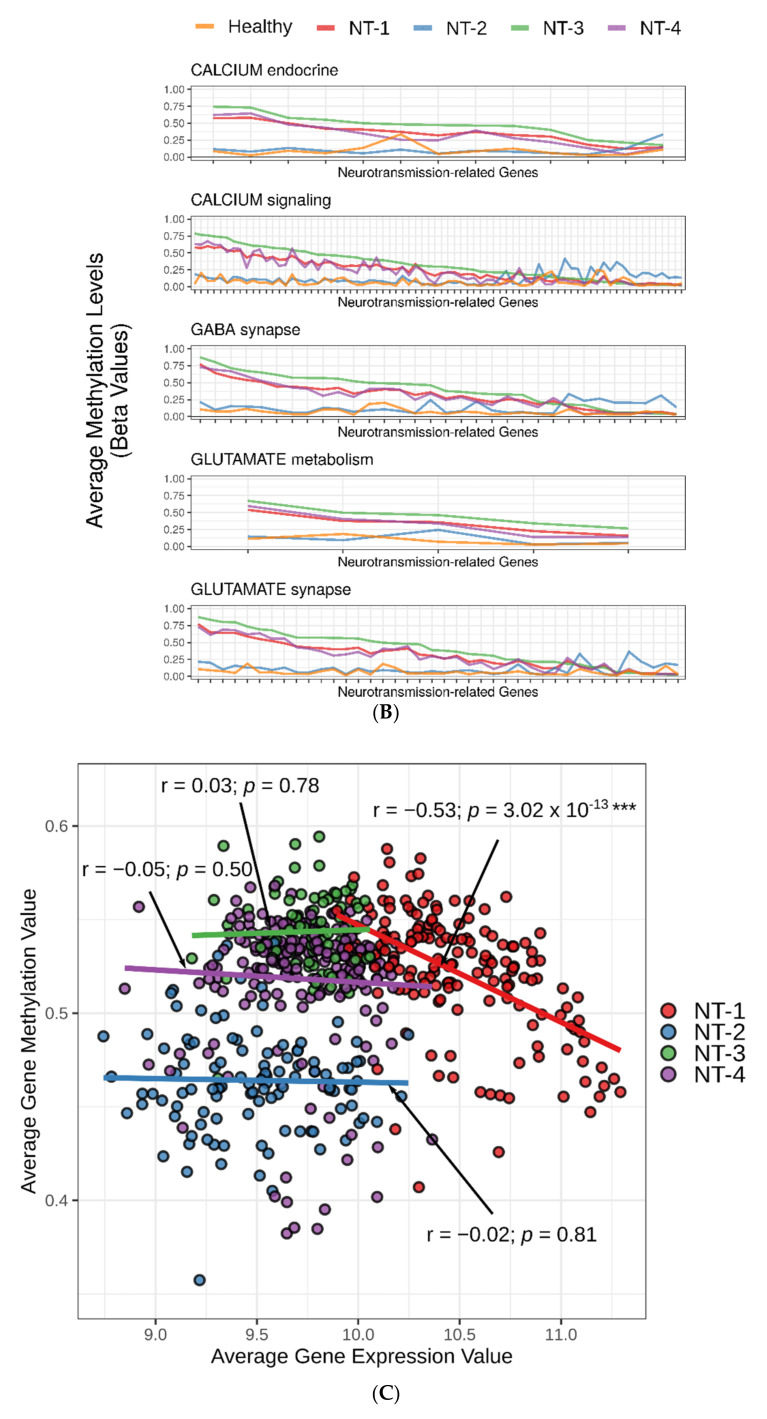
(**A**) DNA methylation levels associated with neurotransmission-related genes used for NT1-4 glioma unsupervised clustering. Average methylation beta values of 351 neurotransmission-related genes reflect DNA hypomethylation (beta = 0) and hypermethylation (beta = 1). Healthy samples (*n* = 2) were also added to the analysis. (**B**) DNA methylation profiles associated with neurotransmission-related genes used for NT1-4 glioma unsupervised clustering. A total of 351 individual gene methylation beta-values are sorted in descending order and rated between 0 and 1. Healthy samples (*n* = 2) were also added to the analysis. (**C**) Correlation between average neurotransmission-related gene DNA hypermethylation and expression in NT-1-4 gliomas (*n* = 661). Each point represents a glioma sample, and the color is specifically related to the NT glioma cluster (*** *p* < 0.001).

We then sought to evaluate the role of DNA methylation, which is an important epigenetic mechanism involved in gliomagenesis, in modulating neurotransmission gene expression in NT gliomas. Using the TCGA methylation quantification dataset, we first examined the DNA methylation levels of GABA-, glutamate- and calcium-related genes used for NT-1-4 glioma unsupervised clustering. For this, we extracted the methylation beta values of the differentially expressed neurotransmission-related genes between NT-1-4 gliomas (Wilcoxon rank sum test Bonferroni-corrected *p*-value less than 0.001 and absolute log2 fold change superior to 1). Methylation beta values equal to 0 and equal to 1 reflect DNA hypomethylation and hypermethylation, respectively. We found that NT-2 gliomas were associated with lower average beta values than NT-1, NT-3 and NT-4 gliomas (Figure 5A, Wilcoxon rank sum test adjusted by Bonferroni correction; *p* = 4.9 × 10^−10^; *p* = 5.8 × 10^−14^; *p* = 1.1 × 10^−5^, respectively). The average beta values of NT-2 were also significantly higher than in healthy samples albeit with lower statistical significance because of the number of healthy samples in the analysis (*n* = 2) (Wilcoxon rank sum test *p* = 1.2 × 10^−9^). We further investigated DNA methylation levels for individual genes ascribed to calcium endocrine, calcium signaling, GABA synapse, glutamate metabolism and glutamate synapse signaling pathways by generating methylation profiles (Figure 5B). Again, NT-2 gliomas and healthy samples were associated with overall DNA hypomethylation. Interestingly, a significant negative correlation between DNA methylation and expression levels was only maintained for NT-1 gliomas (r = −0.53; *p* = 3.02 × 10^−13^; Figure 4D). However, DNA methylation levels in NT-2, NT-3 and NT-4 gliomas correlated weakly with gene expression levels (r = −0.23; *p* = 1.21 × 10^−5^; Figure 5C). 

### 3.6. NT-1 and NT-2 Gliomas Are Regulated by More Complex Epigenetic Mechanisms Involving Differential Expression of microRNA

**Figure 6:**
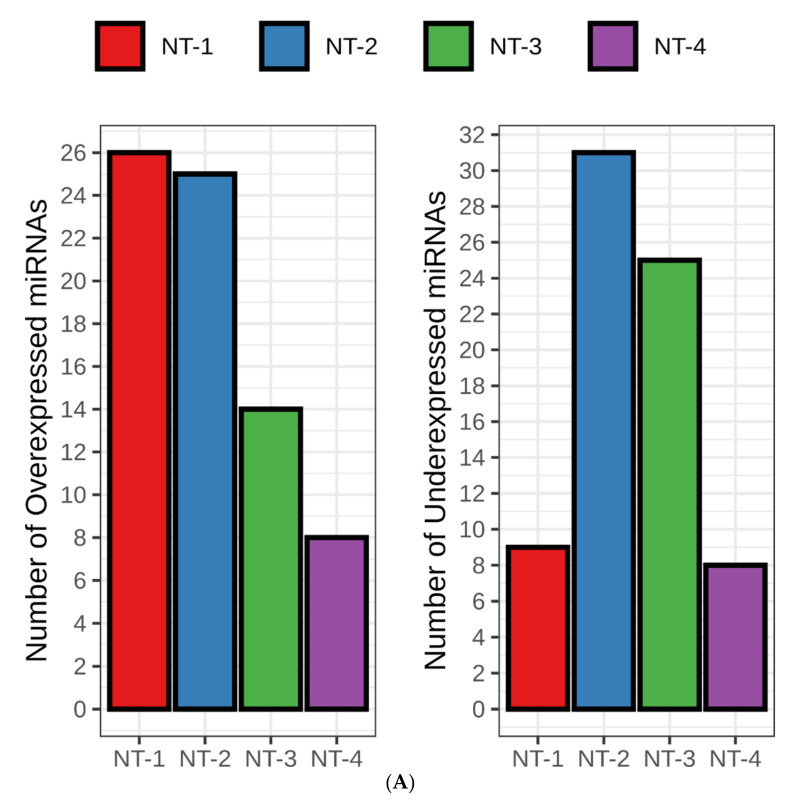

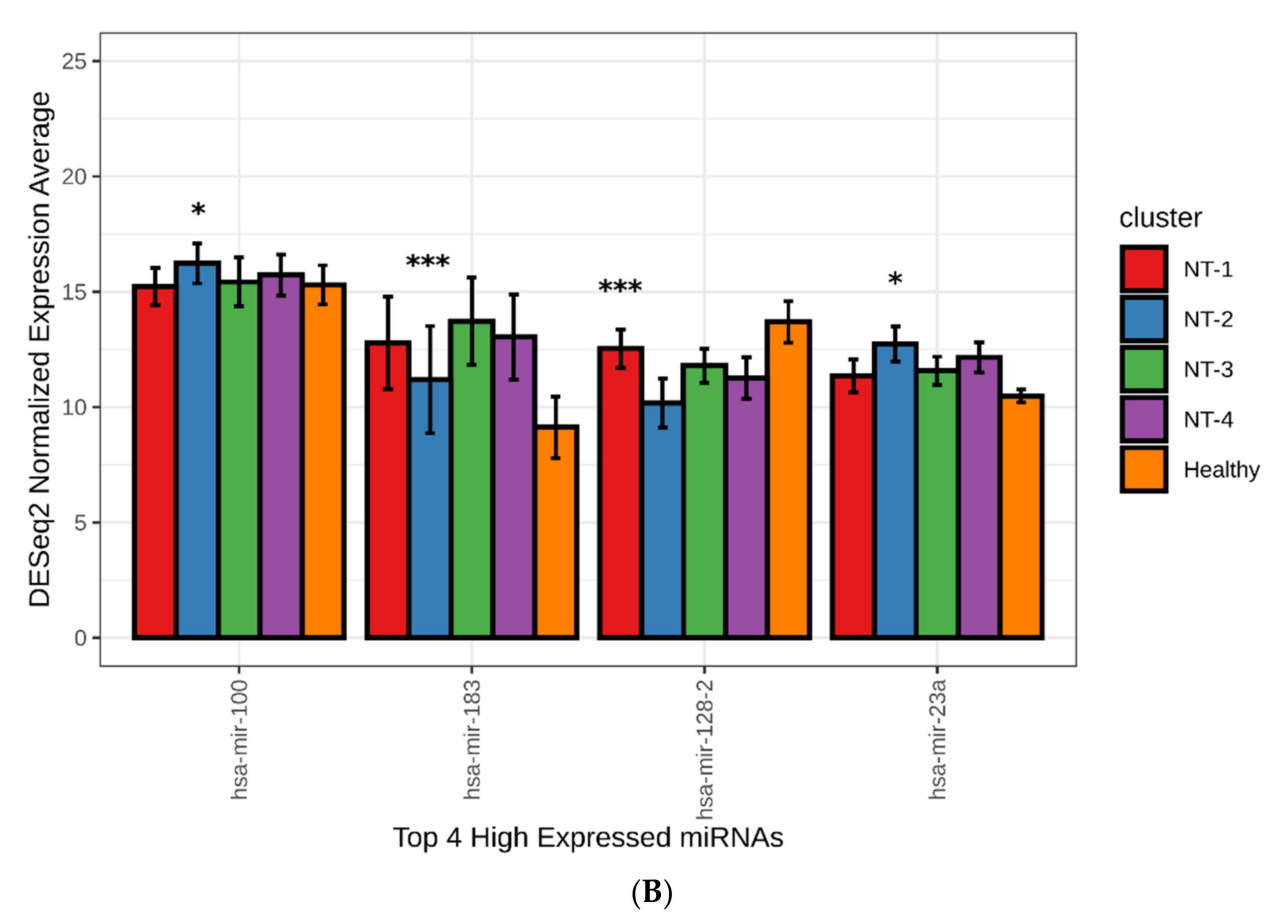
(**A**). Number of differentially highly or lowly expressed miRNAs identified in NT-1-4 gliomas. The differential analysis was performed using DESeq2 R packages without taking into account healthy samples (*n* = 5). High or low expression of miRNAs in an NT cluster indicates the miRNAs having the highest/lowest expression in one of the four NT clusters. (**B**) Average expression levels for hsa-mir-100, hsa-mir-183, hsa-mir-128 and hsa-mir-23a in NT-1-4 gliomas. Stars represent the significance of the glioma cluster compared to the others (healthy samples are excluded in the comparison: * *p* < 0.05; *** *p* < 0.001).

MicroRNAs (miRNAs) are small, single-stranded, non-coding RNA molecules (21–25 nucleotides in length) that play an important role in tumorigenesis through RNA silencing and post-transcriptional regulation of gene expression [31]. We explored their potential regulatory roles on GABA-, glutamate- and calcium-associated gene expression in gliomas by extracting from the RNA Interactome database [27] the miRNAs that interacted directly with our 351 neurotransmission-related genes. We identified 73 differentially expressed miRNAs (DE-miRNAs) in this analysis. The largest counts of highest-expressed DE-miRNAs were found in NT-1 and NT-2 gliomas (26, 25, 14 and 8 DE-miRNAs for NT-1, NT-2, NT-3 and NT-4, respectively; Figure 6A) and the largest counts of lowest-expressed DE-miRNAs were found in NT-2 and NT-3 gliomas (31, 25, 9 and 8 for NT-2, NT-3, NT-1 and NT-4, respectively).

A total of 29 DE-miRNAs were found to be associated with higher expression compared to the average expression of all DE-miRNAs (average of 4.3 DESeq2 normalized counts; Supplementary Data Table S5). Amongst these 29, the top 4 were the miRNAs hsa-mir-100, hsa-mir-183, hsa-mir-128-2 and hsa-mir-23a (Figure 6B and Supplementary Data Table S5). The hsa-mir-100 and hsa-mir-23a mirRNAs were significantly overexpressed in NT-2 gliomas when compared to NT-1, NT-3, and NT-4 clusters (Bonferroni-adjusted *p* < 0.05 for all cluster pairwise comparison). The hsa-mir-128-2 was overexpressed in NT-1 (Bonferroni-adjusted *p* < 10 × 10^−5^ for all cluster pairwise comparison). Expression of hsa-mir-183 was lower in NT-2 gliomas (Bonferroni-adjusted *p* < 10 × 10^−5^ for all cluster pairwise comparison). Overall, neurotransmitter-related miRNAs appeared to be more deregulated in NT-2 gliomas when compared to other glioma subgroups.

### 3.7. Neurotransmission-Related Gene Expression Correlates with the Immune Response Signaling Pathways in NT-1-4 Glioma Clusters

To look into the regulation of specific cellular signaling pathways by neurotransmitter-related genes in glioma, we performed a correlation analysis of the TCGA glioma dataset between previously-identified NT1-4 discriminatory genes (CHRM1, CHRM3 and GRIN1) and non-neurotransmission-related genes. We retrieved 7758 and 7346 genes with negative and positive correlation, respectively (Bonferroni-adjusted *p* < 0.05, Supplementary Data Table S6). 

Analyses of negatively-correlated genes revealed enrichment for genes pertaining to 15 specific cellular pathways such as cell activation, immune effector process, cell population proliferation, response to biotic stimulus, primary metabolic process, symbiotic process, movement of cell or subcellular component, response to external stimulus, etc. (Supplementary Data Figure S3). Amongst these pathways, 10 out of 15, such as cell activation, cell population proliferation and movement of cell and/or subcellular component process groups, were mainly composed of immune cellular processes such as positive regulation of leukocyte activation, T cell activation and leukocyte proliferation (Supplementary Data Figure S4).

Positively-correlated genes were significantly associated with signaling pathways such as system process, macromolecule localization, establishment of localization, regulation of biological quality, cellular component organization or biogenesis and cell communication (Supplementary data Figure S5). In summary, neurotransmission gene expression in gliomas correlates with various cellular pathways related to immunity.

### 3.8. Immune Cell Characterization Reveals Different Tumor Immune Microenvironment Composition

**Figure 7:**
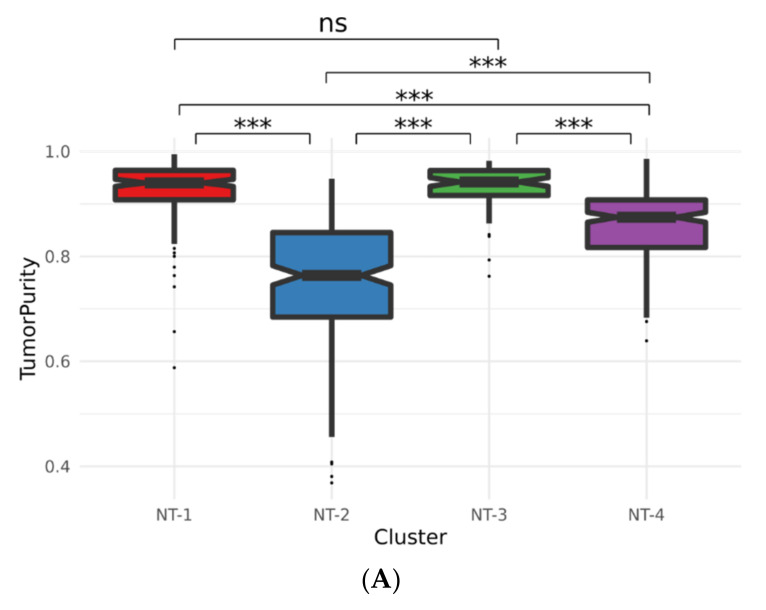

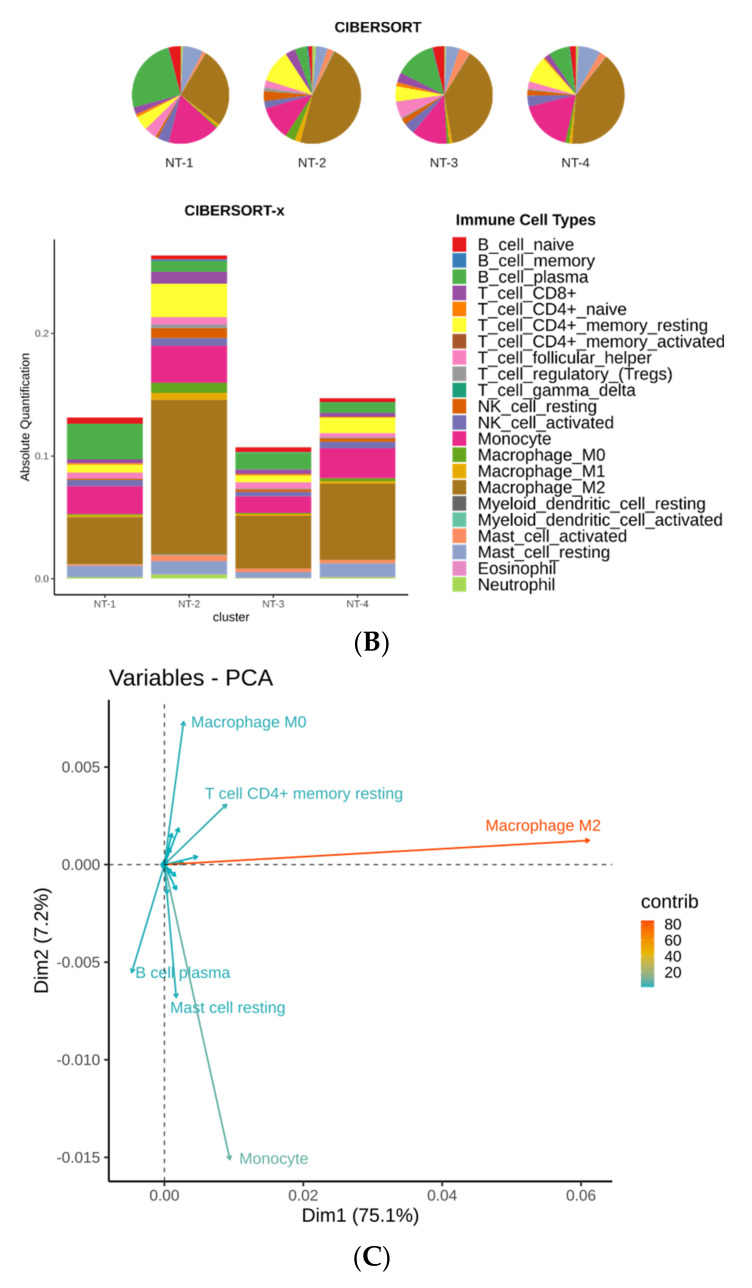
(**A**) ESTIMATE R tumor purity for the NT-1, NT-2, NT-3 and NT-4 gliomas. Purity scores were inferred from the presence of infiltrating immune/stromal cells (*** *p* < 0.001). (**B**) Immune cell type inference for NT-1, NT-2, NT-3 and NT-4 gliomas using CIBERSORT and CIBERSORT-ABS tools. CIBERSORT generates an immune cell fraction relative to the total immune cell content; CIBERSORTx generates an absolute proportion of each cell type. (**C**) Variable contributions within the first and second principal components. The PCA was performed on the CIBERSORTx immune cell composition inference data.

The identification of a high number of immune processes with the CHRM1, CHRM3 and GRIN-1 neurotransmission-related gene signature may suggest that NT-related gliomas possess distinct tumor immune microenvironments. We first performed an ESTIMATE R tumor purity calculation on NT-1-4 gliomas, as this tool evaluates immune and stromal gene expression signatures. We found that the NT-1 gliomas were associated with significantly higher tumor purity scores when compared to the NT-2 (Wilcoxon rank sum test *p* = 3.01 × 10^−45^) and NT-4 (Wilcoxon rank sum test *p* = 5.2 × 10^−26^, Figure 7A) gliomas, reflecting lower levels of immune cells in the NT-1 gliomas. There were no significant differences observed between NT-1 and NT-3 gliomas.

To further substantiate this observation, we also inferred the immune cell type composition of each glioma cluster using CIBERSORT [32] and CIBERSORTx [33] tools (Figure 7B). The CIBERSORTx analysis showed similar absolute immune cell quantification between the NT-1 and NT-4 gliomas (Wilcoxon rank sum test *p* = 0.28). NT-2 gliomas were associated with a higher number of immune cells (Wilcoxon rank sum test *p* = 6.42 × 10^−32^, *p* = 1.94 × 10^−27^ and *p* = 1.70 × 10^−26^ compared to NT-1, NT-3 and NT4, respectively). When analyzing the CIBERSORT results, NT-2, NT-3 and NT-4 gliomas were significantly associated with a higher fraction of M2-phenotype macrophages when compared to NT-1 gliomas (Wilcoxon rank sum test *p* = 3.90 × 10^−30^, *p* = 2.01 × 10^−8^ and *p* = 1.49 × 10^−17^, respectively). In addition, NT-1 gliomas had a higher fraction of plasma B cells (Wilcoxon rank sum test *p* = 1.73 × 10^−42^, *p* = 2.42 × 10^−9^ and *p* = 2.33 × 10^−35^, respectively) when compared to the three other groups. NT-1 gliomas also had a higher fraction of monocytes when compared to NT-2 and NT-3 gliomas (Wilcoxon rank sum test *p* = 7.45 × 10^−10^, *p* = 1.23 × 10^−5^ and *p* = 8.36 × 10^−1^, respectively). Other immune cell types were identified to be significantly enriched in NT-1 gliomas and are described in the Supplementary Data Table S7.

Principal component analysis (PCA) on the CIBERSORTx immune data showed that the first and second components explained 82.3% of the total variability (75.1% and 7.2% for the first and second component, respectively, Figure 7C). The first principal component mainly consists of the M2 macrophage variable (93.87%). Monocytes were the main contributors to the second principal component (59.89%), with macrophages M0 (14.06%), mast cells resting (12.15%) and B cells plasma (8.01%) also contributing.

### 3.9. Neurotransmission-Related Transcriptomic Profiling on the Chinese Glioma Genome Atlas Cohort

**Figure 8:**
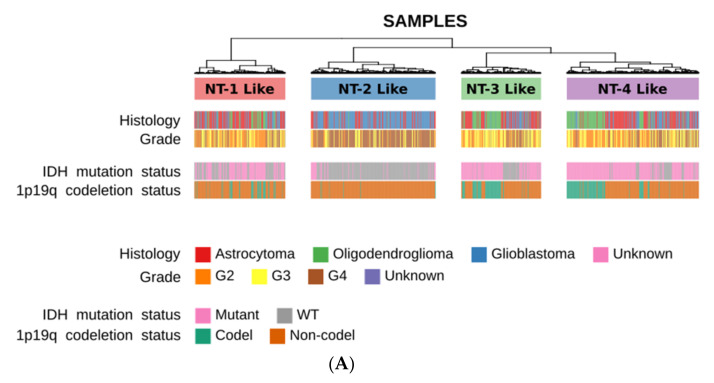

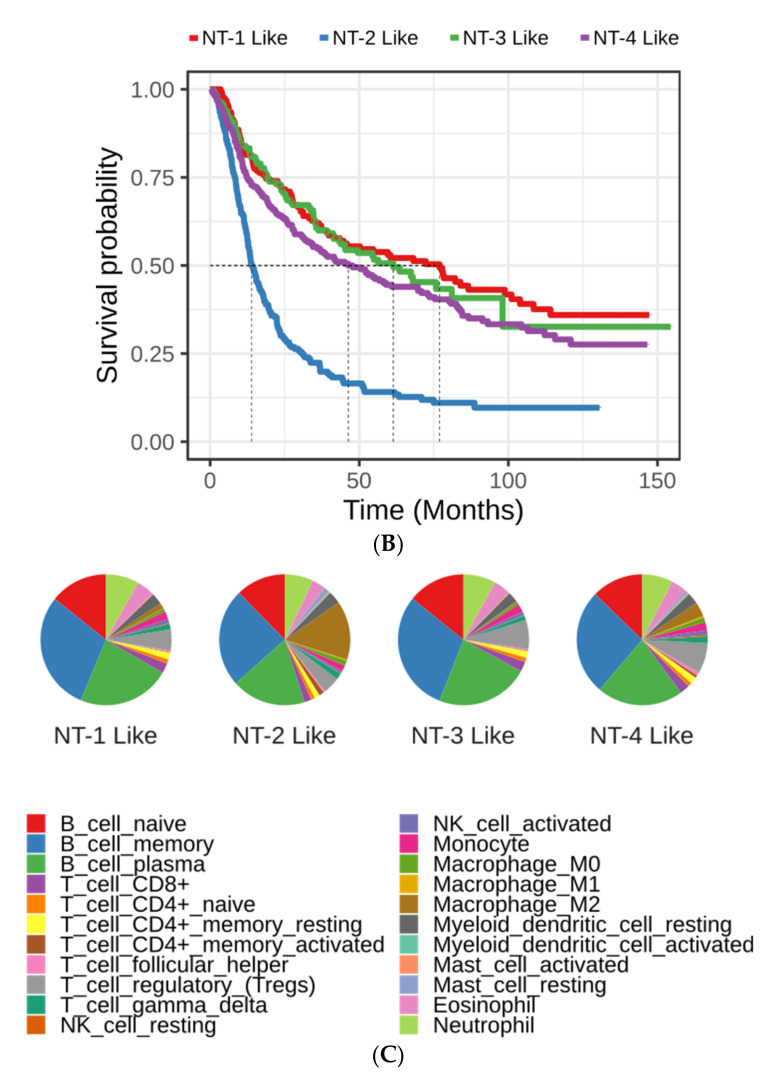
(**A**) Distribution of glioma molecular alterations in CGGA neurotransmission-related glioma clusters. The four clusters were generated by hierarchical unsupervised clustering using Pearson correlation (glioma samples in column) and Minkowski distance (genes in rows). Ward.D2 clustering method was selected for this clustering. (**B**) Kaplan–Meier survival curves associated with the CGGA NT-1-4 like glioma clusters. Red, blue, green and purple represent the various CGGA NT-1-4-like glioma clusters, respectively. Cluster-to-cluster significance was calculated using the log-rank test. (**C**) Immune cell type inference for NT-1-4 like gliomas using CIBERSORT. CIBERSORT infers the immune cell fraction relative to the total immune cell content.

We tested the reproducibility of our findings using 889 glioma samples from the Chinese Glioma Genome Atlas cohort (CGGA) with the neurotransmission-related gene set (351 genes) used in the analysis of the TCGA cohort. We performed unsupervised clustering and generated four different clusters, which were identified as NT-1-like (*n* = 189), NT-2-like (*n* = 259), NT-3-like (*n* = 166) and NT-4-like (*n* = 275) glioma clusters (Figure 8A). Similar to the TCGA cohort, the NT-2-like cluster was mainly composed of IDH-wt gliomas (222/259 or 85.71%) whereas IDH-mutated gliomas were predominant in the NT-3- and NT-4-like clusters (122/166, 73.49% and 226/275, 82.18% for the NT-3- and NT-4-like clusters, respectively; Supplementary Data Table S8). The NT-1-like glioma cluster was composed of a mixture of IDH-mutated (112/189 or 59.26%) and IDH-wt gliomas (77/189 or 40.74%). The 1p/19q chromosomal co-deletion did not predominate in any cluster.

Survival analysis showed a similar survival for NT-1-like, NT-3-like and NT-4-like clusters (log-rank test *p* > 0.05 Figure 8B). NT-2-like gliomas had the shortest survival rate when compared to the three other clusters (log-rank test, *p* = 8.76 × 10^−19^, *p*= 1.02 × 10^−16^ and *p* = 3.66 × 10^−15^ when compared with NT-1-like, NT-3-like and NT-4-like, respectively).

In terms of immune cell type composition, NT-2-like gliomas were associated with a higher cell fraction of M2-phenotype macrophages, similar to the TCGA cohort analysis findings. (Wilcoxon rank sum test *p* = 4.76 × 10^−22^, *p* = 1.03 × 10^−29^ and *p* = 2.09 × 10^−17^ compared to the NT-1-like, NT-3-like and NT4-like, respectively; Figure 8C). In general terms and similar to the TCGA analyses, neurotransmission-based unsupervised clustering of the CGGA glioma expression dataset recapitulated four subgroups with similar survival and inflammatory microenvironment characteristics.

## 4. Discussion

Current glioma therapies lead to limited improvement in median overall survival in patients with high-grade infiltrating gliomas [34]. Beyond targeting tumor cells, there is currently a shift of focus towards understanding components of the tumor microenvironment, such as surrounding neural cells (neurons and glia), hematopoietic cells (monocyte/macrophage/microglia and T cells) or blood vessels, in an attempt to overcome redundant compensatory mechanisms [35]. In this large-scale multi-omics analysis, IDH-wildtype and IDH-mutated infiltrating gliomas were studied through the lens of neurotransmission-related (GABA, glutamate and calcium) gene expression patterns with the aim of unraveling specific vulnerabilities and cellular pathways.

Neurotransmission-based unsupervised clustering enabled the proper classification of the majority of infiltrating gliomas into current WHO tumor categories (IDH-wt gliomas, IDH-mutated, 1p/19q oligodendrogliomas and IDH-mutated astrocytomas), suggesting that neurotransmission-related pathways are differentially regulated in tumor cells and/or their microenvironment according to tumor subtype, and also reaffirming the importance of IDH1/2 mutations and of 1p/19q-codeletion for glioma stratification. Interestingly, this strategy also identified a novel NT-1 glioma subgroup mostly comprised of IDH-mutated gliomas, which also included “normal-like” IDH-wt gliomas associated with a longer survival [1,29].

The NT-1 subgroup primarily distinguishes itself by its overexpression of GABA, glutamate and calcium genes. Conversely, the NT-2 cluster, which is principally comprised of more-aggressive IDH-wildtype glioblastomas, is associated with a lower expression of neurotransmission-related genes. IDH-mutated gliomas with (NT-3) or without (NT-4) the 1p/19q codeletion show intermediate levels of expression. This supports the importance of the IDH mutation in regulating neurotransmission-related gene programs in glioma [36,37].

The role of the IDH mutation as a trigger of the CpG island methylator phenotype in IDH-mutated gliomas via the production of 2-hydroxyglutarate (2-HG) oncometabolite is well-established [38,39]. It is interesting to note that—while the average methylation of neurotransmission genes in NT-1 gliomas is high, as expected from a group mainly composed of IDH-mutated tumors—these tumors are distinct from other IDH-mutated gliomas by the partial preservation of their capacity to silence neurotransmission genes through methylation. We can speculate that epigenetic DNA methylation events that follow IDH mutation in early gliomagenesis target neurotransmission genes randomly, thereby accounting for heterogeneous neurotransmission-related profiles and selective vulnerabilities within IDH-mutated tumors. Neurotransmitter-related genes amenable to epigenetic reprogramming may impact the therapeutic efficacy of experimental anti-cancer DNA demethylating drugs [40].

Neurotransmission-based glioma clusters are also distinct by virtue of altered expression of miRNAs interacting with GABA-, glutamate- and calcium-related genes. In particular, four miRNAs (hsa-mir-100, hsa-mir-183, hsa-mir-128-2 and hsa-mir-23a) were shown to be highly expressed in comparison to the other deregulated miRNAs. In gliomas, hsa-mir-183 promotes cell proliferation, invasion, angiogenesis and radioresistance [41,42,43,44,45,46] and is overexpressed in NT-3 and NT-4 IDH-mutated gliomas. The hsa-mir-128-2 miRNA, which is overexpressed in the NT-1 cluster, has an inhibitory role on tumor growth and angiogenesis in glioma [47]. Furthermore, its overexpression is associated with an increase of temozolomide cytotoxicity and chemosensitivity in glioma and a decrease in chemotherapeutic resistance in breast cancer [48,49,50]. Next, hsa-mir-23a promotes cell growth [51], proliferation [52] and invasion [53] in glioma and participates in colorectal cancer cell chemoresistance [54,55]. As for hsa-mir-100, its overexpression is associated with reductions in cell proliferation [56], growth [57] and chemoresistance [58] even though it is overexpressed in more aggressive NT-2 gliomas. Overall, the differential expression of miRNAs directly interacting with neurotransmission-related genes represents another layer of epigenetic diversity between NT-1-4 gliomas that may impact treatment response and resistance.

Correlations between the CHRM1, CHRM3 and GRIN-1 NT-1-4 glioma intersecting gene signatures and various immune signaling pathways suggested that NT gliomas may be endowed with distinct tumor immune microenvironments. Further, 2-HG is an important mediator of tumor immunity in IDH-mutated gliomas. It acts as a suppressor of antitumor T-cell activity and also impedes macrophage recruitment in gliomas by altering tryptophan metabolism [59,60]. Lower pools of M2-phenotype macrophages were detected in NT-1 gliomas when compared to NT-2-4 gliomas. Considering the anti-tumor and immunosuppressive functions of this type of macrophage in gliomas, this observation could be in keeping with a less suppressive and less tumor-supportive inflammatory microenvironment in NT-1 gliomas, partly explained by their IDH status but also promoted by operational neurotransmitter signaling pathways modulated by released GABA or glutamate in cancer or microenvironment cells [61].

We identified novel, neurotransmission-based glioma subgroups with their peculiarities in terms of expression, epigenetics (methylation and miRNAs) and inflammatory microenvironment. These findings may be of clinical relevance should neurotransmitter pathways impacting tumor aggressiveness be actionable or reactivable in gliomas, whether it is through epigenetic-based strategies for IDH-mutated tumors or any other strategy for IDH-wt gliomas.

The adult gliomas included in this study are infiltrative by nature. It is thus expected that brain biopsy samples will include non-neoplastic brain cells. We do expect that some of the differentially expressed NT genes identified in this study belong to the non-neoplastic tumor microenvironment, where they can still impact tumor aggressiveness. Further bioinformatics studies on microdissected tumor tissue and single-cell RNA sequencing data will help to specify more precisely the location of neurotransmission targets in tumor cells versus non-neoplastic tumor cells of the microenvironment. Experimental studies targeting neurotransmitter signaling elements and relevant miRNAs in vitro will also further our knowledge regarding the impact of these pathways on glioma aggressiveness.

## 5. Conclusions

This multi-omics analysis revealed the existence of neurotransmission-based glioma categories with significant differences in regard to neurotransmission-related gene expression, methylation, and miRNA profiles in adult gliomas. It also revealed alterations in the nature of the tumor inflammatory microenvironment between NT glioma subgroups. Deciphering operational neurotransmitter signaling pathways and underpinning mechanisms that may represent actionable targets is a promising personalized treatment avenue to explore for glioma patients as a complement to current radiotherapy and chemotherapy treatments in an attempt to improve clinical outcomes.

## Figures and Tables

**Table 1:** Age at diagnosis and gender for NT-1-4 gliomas.

Variable	Category	NT-1 (*n* = 168)	NT-2 (*n* = 188)	NT-3 (*n* = 81)	NT-4 (*n* = 224)
Gender	Female	77 (45.8%)	75 (39.9%)	39 (48.1%)	88 (39.3%)
	Male	90 (53.6%)	113 (60.1%)	42 (51.9%)	135 (60.3%)
	Unknown	1 (0.6%)	0 (0.0%)	0 (0.0%)	1 (0.4%)
Age at Diagnosis	Min.	14	24	22	18
	1st Qu.	32.5	51	36	31
	Median	40	59	46	38
	Mean	42.69	58.12	45.59	41.03
	3rd Qu.	53	66.25	53	49
	Max.	87	85	75	89

**Table 2:** Univariate and multivariate Cox regression analysis. Cluster (NT-1, NT-2, NT-3 and NT-4), gender (male and female), age at diagnosis and glioma grade (G1, G2, G3 and G4) information were integrated into this analysis as covariates.

		Univariate Cox Regression			Multivariate Cox Regression		
Covariate	Category	Beta	HR (95% CI for HR)	*p*-value	Beta	HR (95% CI for HR)	*p-*value
Cluster	NT-1	Reference			Reference		
	NT-2	2.216	9.169 (6.167–13.633)	6.62 × 10^−28^	0.901	2.461 (1.533–3.952)	1.93 × 10^−4^
	NT-3	−0.262	0.769 (0.398–1.485)	4.35 × 10^−1^	−0.456	0.634 (0.327–1.229)	1.77 × 10^−1^
	NT-4	0.433	1.543 (1.020–2.333)	4.00 × 10^−2^	0.31	1.363 (0.894–2.079)	1.51 × 10^−1^
Gender	Female	Reference			Reference		
	Male	0.202	1.224 (0.947–1.582)	1.22 × 10^−1^	0.029	1.029 (0.794–1.334)	8.29 × 10^−1^
Age at Diagnosis		0.066	1.068 (1.058–1.079)	9.31 × 10^−42^	0.039	1.040 (1.028–1.052)	4.32 × 10^−11^
Grade	G2	Reference			Reference		
	G3	1.148	3.153 (2.054–4.841)	1.53 × 10^−7^	0.972	2.644 (1.717–4.074)	1.03 × 10^−5^
	G4	2.976	19.605 (12.821–29.978)	6.38 × 10^−43^	1.772	5.883 (3.534–9.791)	9.30 × 10^−12^
	Unknown	1.092	2.979 (1.652–5.370)	2.83 × 10^−4^	0.92	2.510 (1.381–4.565)	2.55 × 10^−3^

## Data Availability

The data used in the study are available in the TCGA public databases. The datasets supporting the conclusions of this article are included within the article (and its additional files).

## Supplementary Materials

The following are available online at https://www.mdpi.com/article/10.3390/jpm12040633/s1. Supplementary Data Figure S1: Upset plot describing the 351 neurotransmission-related genes used in our analyses. These gene sets were taken from the KEGG metabolic database, Supplementary data Table S1: List of 351 neurotransmitter-related genes used for unsupervised clustering (>10 counts), Supplementary Data Figure S2: Average entropy of the TCGA glioma expression dataset unsupervised clustering based on the IDH and 1p/19q codeletion status, according to the number of clusters generated. The optimal number of clusters is equal to 4 (red dot), Supplementary Data Table S2: Distribution of the NT-1-4 glioma clusters among the 661 TCGA glioma samples, Supplementary Data Figure S3: Gene ontology enrichment analysis on correlated genes between previously-identified NT1-4 intersecting genes (CHRM1, CHRM3 and GRIN1) and non-neurotransmission-related genes., Supplementary Data Table S3: Distribution of biomolecular and epidemiological variables among NT-1-4 glioma clusters, Supplementary Data Figure S4: Significant Gene Ontology group terms associated with cell activation, cell proliferation and movement of cell or subcellular component gene ontology level 3 gene ontology group terms. Colors represent the correlation type (blue and red for negative and positive correlations, respectively). Supplementary Data Table S4: Distribution of overexpressed and underexpressed neurotransmission-related genes in NT-1-4 gliomas, Supplementary Data Figure S5. Significant Gene Ontology group terms associated with system process, macromolecule localization, establishment of localization, regulation of biological quality, cellular component organization or biogenesis and cell communication gene level 3 gene ontology group terms. Colors represent the correlation type (blue and red for negative and positive correlations, respectively), Supplementary Data Table S5: Average expression levels of 73 differentially expressed miRNAs in NT-1-4 gliomas. Supplementary Data Table S6: Positive and negative correlations between CHRM1, CHRM3 and GRIN-1 genes and nonrelated neurotransmission genes. Supplementary Data Table S7: Comparison of immune cell type enrichment among NT-1-4 glioma clusters generated by the CIBERSORT analysis tool. Supplementary Data Table S8: Distribution of biomolecular and epidemiological variables among CGGA NT-1-4-like glioma clusters.
