# Supplementary material for: Deciphering of Adult Glioma Vulnerabilities through Expression Pattern Analysis of GABA, Glutamate and Calcium Neurotransmitter Genes
Source: J Pers Med. 2022 Apr 14;12(4):633. doi: 10.3390/jpm12040633 (PMC9030730; doi:10.3390/jpm12040633)
Supplement: Supplementary file 1 [file jpm-12-00633-s001.zip › supplementary_data_figureS4_negative_correlation_goterm.pdf]

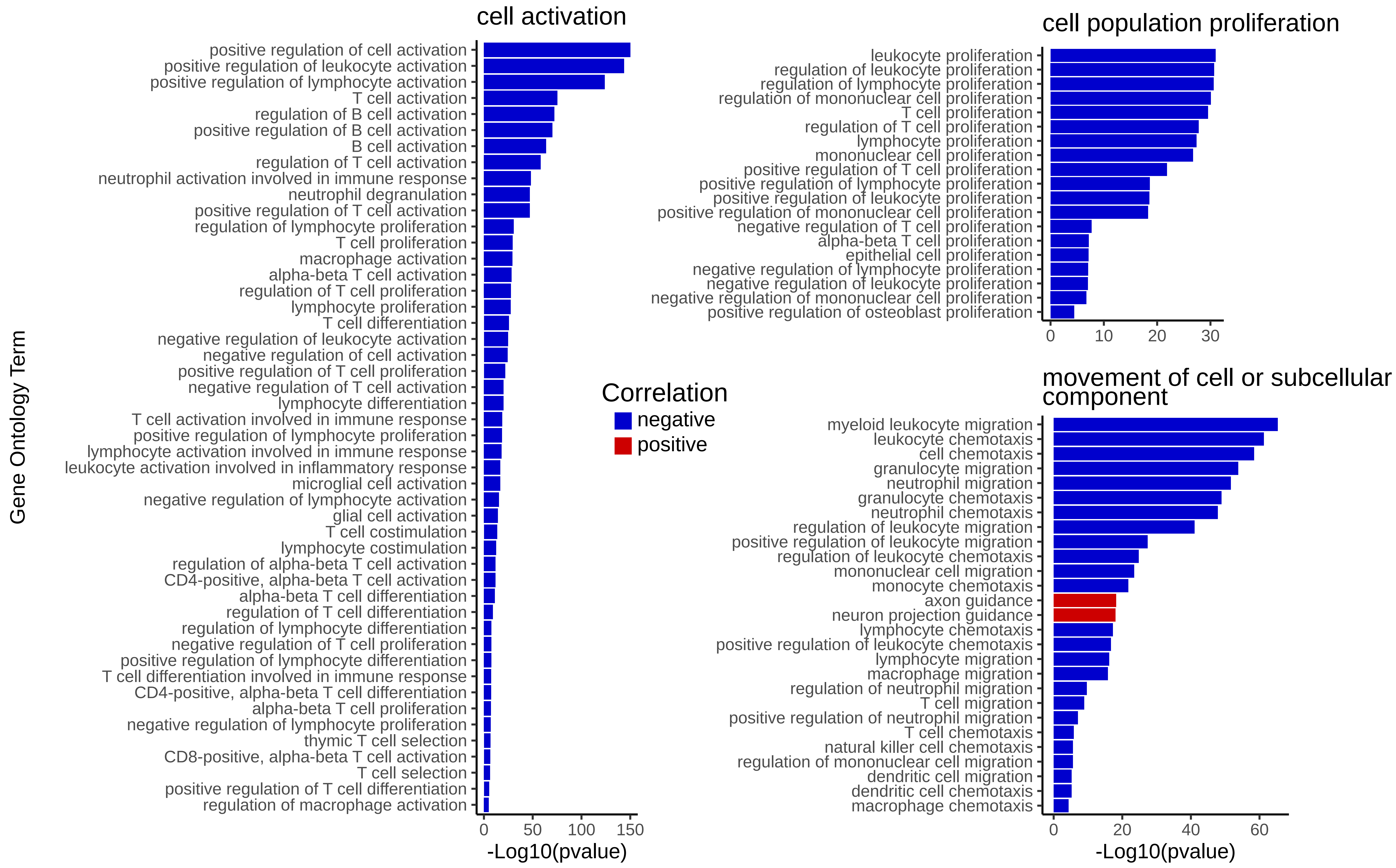

cell activation
positive regulation of cell activation
positive regulation of leukocyte activation
positive regulation of lymphocyte activation
T cell activation
regulation of B cell activation
positive regulation of B cell activation
B cell activation
regulation of T cell activation
neutrophil activation involved in immune response
neutrophil degranulation
positive regulation of T cell activation
regulation of lymphocyte proliferation
T cell proliferation
macrophage activation
alpha-beta T cell activation
regulation of T cell proliferation
lymphocyte proliferation
T cell differentiation
negative regulation of leukocyte activation
negative regulation of cell activation
positive regulation of T cell proliferation
negative regulation of T cell activation
lymphocyte differentiation
T cell activation involved in immune response
positive regulation of lymphocyte proliferation
lymphocyte activation involved in immune response
leukocyte activation involved in inflammatory response
microglial cell activation
negative regulation of lymphocyte activation
glial cell activation
T cell costimulation
lymphocyte costimulation
regulation of alpha-beta T cell activation
CD4-positive, alpha-beta T cell activation
alpha-beta T cell differentiation
regulation of T cell differentiation
regulation of lymphocyte differentiation
negative regulation of T cell proliferation
positive regulation of lymphocyte differentiation
T cell differentiation involved in immune response
CD4-positive, alpha-beta T cell differentiation
alpha-beta T cell proliferation
negative regulation of lymphocyte proliferation
thymic T cell selection
CD8-positive, alpha-beta T cell activation
T cell selection
positive regulation of T cell differentiation
regulation of macrophage activation
0
50
100
150
-Log10(pvalue)
Gene Ontology Term
Correlation
negative
positive
cell population proliferation
leukocyte proliferation
regulation of leukocyte proliferation
regulation of lymphocyte proliferation
regulation of mononuclear cell proliferation
T cell proliferation
regulation of T cell proliferation
lymphocyte proliferation
mononuclear cell proliferation
positive regulation of T cell proliferation
positive regulation of lymphocyte proliferation
positive regulation of leukocyte proliferation
positive regulation of mononuclear cell proliferation
negative regulation of T cell proliferation
alpha-beta T cell proliferation
epithelial cell proliferation
negative regulation of lymphocyte proliferation
negative regulation of leukocyte proliferation
negative regulation of mononuclear cell proliferation
positive regulation of osteoblast proliferation
0
10
20
30
movement of cell or subcellular component
myeloid leukocyte migration
leukocyte chemotaxis
cell chemotaxis
granulocyte migration
neutrophil migration
granulocyte chemotaxis
neutrophil chemotaxis
regulation of leukocyte migration
positive regulation of leukocyte migration
regulation of leukocyte chemotaxis
mononuclear cell migration
monocyte chemotaxis
axon guidance
neuron projection guidance
lymphocyte chemotaxis
positive regulation of leukocyte chemotaxis
lymphocyte migration
macrophage migration
regulation of neutrophil migration
T cell migration
positive regulation of neutrophil migration
T cell chemotaxis
natural killer cell chemotaxis
regulation of mononuclear cell migration
dendritic cell migration
dendritic cell chemotaxis
macrophage chemotaxis
0
20
40
60
-Log10(pvalue)
