# Supplementary material for: Deciphering of Adult Glioma Vulnerabilities through Expression Pattern Analysis of GABA, Glutamate and Calcium Neurotransmitter Genes
Source: J Pers Med. 2022 Apr 14;12(4):633. doi: 10.3390/jpm12040633 (PMC9030730; doi:10.3390/jpm12040633)
Supplement: Supplementary file 1 [file jpm-12-00633-s001.zip › supplementary_data_figureS5_positive_correlation_goterm.pdf]

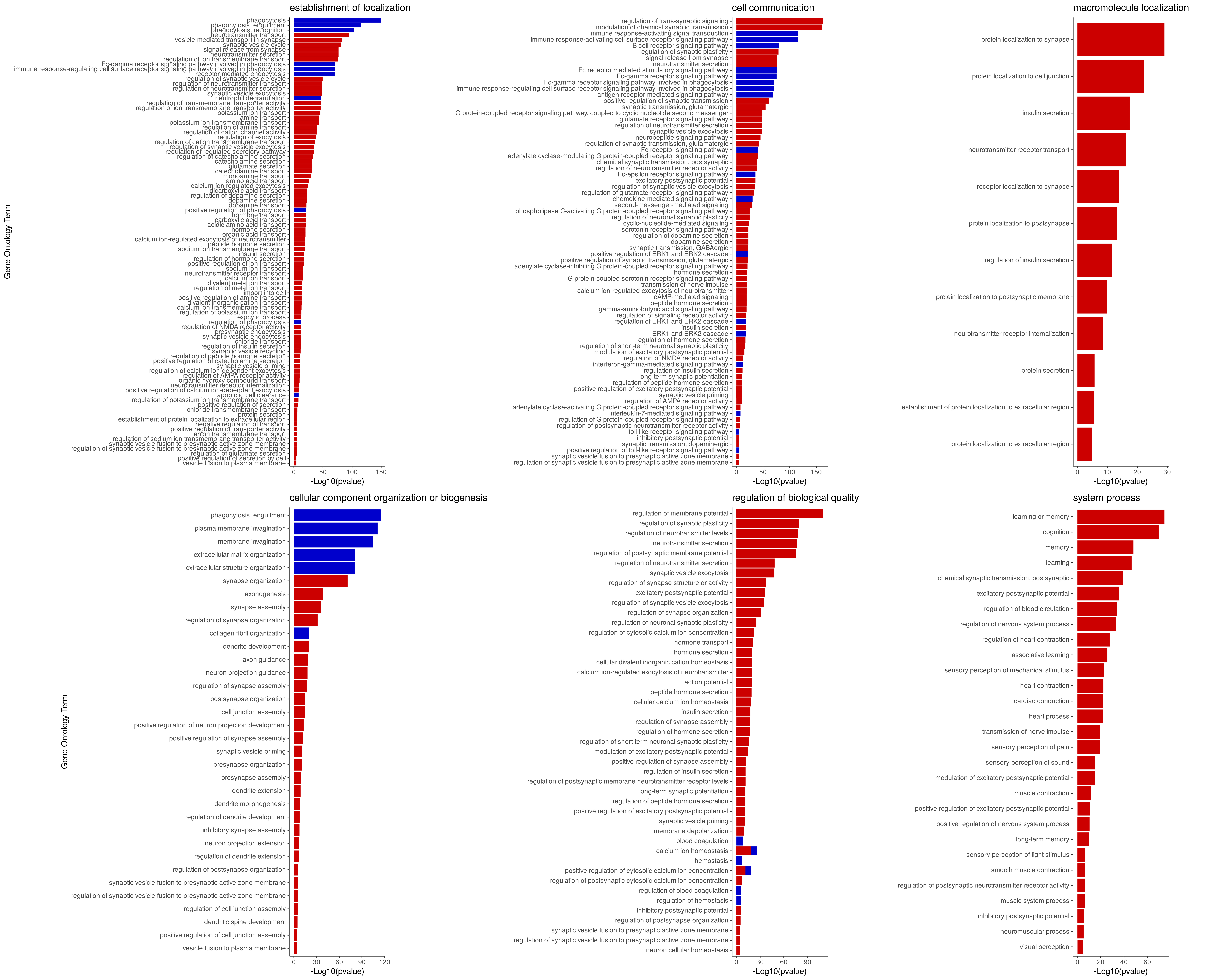
establishment of localization
phagocytosis
phagocytosis, engulfment
phagocytosis, recognition
neurotransmitter transport
vesicle-mediated transport in synapse
synaptic vesicle cycle
signal release from synapse
neurotransmitter secretion
regulation of ion transmembrane transport
Fc-gamma receptor signaling pathway involved in phagocytosis
immune response-regulating cell surface receptor signaling pathway involved in phagocytosis
receptor-mediated endocytosis
regulation of synaptic vesicle cycle
regulation of neurotransmitter transport
regulation of neurotransmitter secretion
synaptic vesicle exocytosis
neutrophil degranulation
regulation of transmembrane transporter activity
regulation of ion transmembrane transporter activity
potassium ion transport
amine transport
potassium ion transmembrane transport
regulation of amine transport
regulation of cation channel activity
regulation of exocytosis
regulation of cation transmembrane transport
regulation of synaptic vesicle exocytosis
regulation of regulated secretory pathway
regulation of catecholamine secretion
catecholamine secretion
glutamate secretion
catecholamine transport
monoamine transport
amino acid transport
calcium-ion regulated exocytosis
dicarboxylic acid transport
regulation of dopamine secretion
dopamine secretion
dopamine transport
positive regulation of phagocytosis
hormone transport
carboxylic acid transport
acidic amino acid transport
hormone secretion
organic acid transport
calcium ion-regulated exocytosis of neurotransmitter
peptide hormone secretion
sodium ion transmembrane transport
insulin secretion
regulation of hormone secretion
positive regulation of ion transport
sodium ion transport
neurotransmitter receptor transport
calcium ion transport
divalent metal ion transport
regulation of metal ion transport
import into cell
positive regulation of amine transport
divalent inorganic cation transport
calcium ion transmembrane transport
regulation of potassium ion transport
exocytic process
regulation of phagocytosis
regulation of NMDA receptor activity
presynaptic endocytosis
synaptic vesicle endocytosis
chloride transport
regulation of insulin secretion
synaptic vesicle recycling
regulation of peptide hormone secretion
positive regulation of catecholamine secretion
synaptic vesicle priming
regulation of calcium ion-dependent exocytosis
regulation of AMPA receptor activity
organic hydroxy compound transport
neurotransmitter receptor internalization
positive regulation of calcium ion-dependent exocytosis
apoptotic cell clearance
regulation of potassium ion transmembrane transport
positive regulation of secretion
chloride transmembrane transport
protein secretion
establishment of protein localization to extracellular region
negative regulation of transport
positive regulation of transporter activity
anion transmembrane transport
regulation of sodium ion transmembrane transporter activity
synaptic vesicle fusion to presynaptic active zone membrane
regulation of synaptic vesicle fusion to presynaptic active zone membrane
regulation of glutamate secretion
positive regulation of secretion by cell
vesicle fusion to plasma membrane
0 50 100 150
-Log10(pvalue)
Gene Ontology Term
cell communication
regulation of trans-synaptic signaling
modulation of chemical synaptic transmission
immune response-activating signal transduction
immune response-activating cell surface receptor signaling pathway
B cell receptor signaling pathway
regulation of synaptic plasticity
signal release from synapse
neurotransmitter secretion
Fc receptor mediated stimulatory signaling pathway
Fc-gamma receptor signaling pathway
Fc-gamma receptor signaling pathway involved in phagocytosis
immune response-regulating cell surface receptor signaling pathway involved in phagocytosis
antigen receptor-mediated signaling pathway
positive regulation of synaptic transmission
synaptic transmission, glutamatergic
G protein-coupled receptor signaling pathway, coupled to cyclic nucleotide second messenger
glutamate receptor signaling pathway
regulation of neurotransmitter secretion
synaptic vesicle exocytosis
neuropeptide signaling pathway
regulation of synaptic transmission, glutamatergic
Fc receptor signaling pathway
adenylate cyclase-modulating G protein-coupled receptor signaling pathway
chemical synaptic transmission, postsynaptic
regulation of neurotransmitter receptor activity
Fc-epsilon receptor signaling pathway
excitatory postsynaptic potential
regulation of synaptic vesicle exocytosis
regulation of glutamate receptor signaling pathway
chemokine-mediated signaling pathway
second-messenger-mediated signaling
phospholipase C-activating G protein-coupled receptor signaling pathway
regulation of neuronal synaptic plasticity
cyclic-nucleotide-mediated signaling
serotonin receptor signaling pathway
regulation of dopamine secretion
dopamine secretion
synaptic transmission, GABAergic
positive regulation of ERK1 and ERK2 cascade
positive regulation of synaptic transmission, glutamatergic
adenylate cyclase-inhibiting G protein-coupled receptor signaling pathway
hormone secretion
G protein-coupled serotonin receptor signaling pathway
transmission of nerve impulse
calcium ion-regulated exocytosis of neurotransmitter
cAMP-mediated signaling
peptide hormone secretion
gamma-aminobutyric acid signaling pathway
regulation of signaling receptor activity
regulation of ERK1 and ERK2 cascade
insulin secretion
ERK1 and ERK2 cascade
regulation of hormone secretion
regulation of short-term neuronal synaptic plasticity
modulation of excitatory postsynaptic potential
regulation of NMDA receptor activity
interferon-gamma-mediated signaling pathway
regulation of insulin secretion
long-term synaptic potentiation
regulation of peptide hormone secretion
positive regulation of excitatory postsynaptic potential
synaptic vesicle priming
regulation of AMPA receptor activity
adenylate cyclase-activating G protein-coupled receptor signaling pathway
interleukin-7-mediated signaling pathway
regulation of G protein-coupled receptor signaling pathway
regulation of postsynaptic neurotransmitter receptor activity
toll-like receptor signaling pathway
inhibitory postsynaptic potential
synaptic transmission, dopaminergic
positive regulation of toll-like receptor signaling pathway
synaptic vesicle fusion to presynaptic active zone membrane
regulation of synaptic vesicle fusion to presynaptic active zone membrane
0 50 100 150
-Log10(pvalue)
macromolecule localization
protein localization to synapse
protein localization to cell junction
insulin secretion
neurotransmitter receptor transport
receptor localization to synapse
protein localization to postsynapse
regulation of insulin secretion
protein localization to postsynaptic membrane
neurotransmitter receptor internalization
protein secretion
establishment of protein localization to extracellular region
protein localization to extracellular region
0 10 20 30
-Log10(pvalue)
cellular component organization or biogenesis
phagocytosis, engulfment
plasma membrane invagination
membrane invagination
extracellular matrix organization
extracellular structure organization
synapse organization
axonogenesis
synapse assembly
regulation of synapse organization
collagen fibril organization
dendrite development
axon guidance
neuron projection guidance
regulation of synapse assembly
postsynapse organization
cell junction assembly
positive regulation of neuron projection development
positive regulation of synapse assembly
synaptic vesicle priming
presynapse organization
presynapse assembly
dendrite extension
dendrite morphogenesis
regulation of dendrite development
inhibitory synapse assembly
neuron projection extension
regulation of dendrite extension
regulation of postsynapse organization
synaptic vesicle fusion to presynaptic active zone membrane
regulation of synaptic vesicle fusion to presynaptic active zone membrane
regulation of cell junction assembly
dendritic spine development
positive regulation of cell junction assembly
vesicle fusion to plasma membrane
0 30 60 90 120
-Log10(pvalue)
Gene Ontology Term
regulation of biological quality
regulation of membrane potential
regulation of synaptic plasticity
regulation of neurotransmitter levels
neurotransmitter secretion
regulation of postsynaptic membrane potential
regulation of neurotransmitter secretion
synaptic vesicle exocytosis
regulation of synapse structure or activity
excitatory postsynaptic potential
regulation of synaptic vesicle exocytosis
regulation of synapse organization
regulation of neuronal synaptic plasticity
regulation of cytosolic calcium ion concentration
hormone transport
hormone secretion
cellular divalent inorganic cation homeostasis
calcium ion-regulated exocytosis of neurotransmitter
action potential
peptide hormone secretion
cellular calcium ion homeostasis
insulin secretion
regulation of synapse assembly
regulation of hormone secretion
regulation of short-term neuronal synaptic plasticity
modulation of excitatory postsynaptic potential
positive regulation of synapse assembly
regulation of insulin secretion
regulation of postsynaptic membrane neurotransmitter receptor levels
long-term synaptic potentiation
regulation of peptide hormone secretion
positive regulation of excitatory postsynaptic potential
synaptic vesicle priming
membrane depolarization
blood coagulation
calcium ion homeostasis
hemostasis
positive regulation of cytosolic calcium ion concentration
regulation of postsynaptic cytosolic calcium ion concentration
regulation of blood coagulation
regulation of hemostasis
inhibitory postsynaptic potential
regulation of postsynapse organization
synaptic vesicle fusion to presynaptic active zone membrane
regulation of synaptic vesicle fusion to presynaptic active zone membrane
neuron cellular homeostasis
0 30 60 90
-Log10(pvalue)
system process
learning or memory
cognition
memory
learning
chemical synaptic transmission, postsynaptic
excitatory postsynaptic potential
regulation of blood circulation
regulation of nervous system process
regulation of heart contraction
associative learning
sensory perception of mechanical stimulus
heart contraction
cardiac conduction
heart process
transmission of nerve impulse
sensory perception of pain
sensory perception of sound
modulation of excitatory postsynaptic potential
muscle contraction
positive regulation of excitatory postsynaptic potential
positive regulation of nervous system process
long-term memory
sensory perception of light stimulus
smooth muscle contraction
regulation of postsynaptic neurotransmitter receptor activity
muscle system process
inhibitory postsynaptic potential
neuromuscular process
visual perception
0 20 40 60
-Log10(pvalue)
