# Supplementary figures and images for: Deciphering of Adult Glioma Vulnerabilities through Expression Pattern Analysis of GABA, Glutamate and Calcium Neurotransmitter Genes
Source: J Pers Med. 2022 Apr 14;12(4):633. doi: 10.3390/jpm12040633 (PMC9030730; doi:10.3390/jpm12040633)

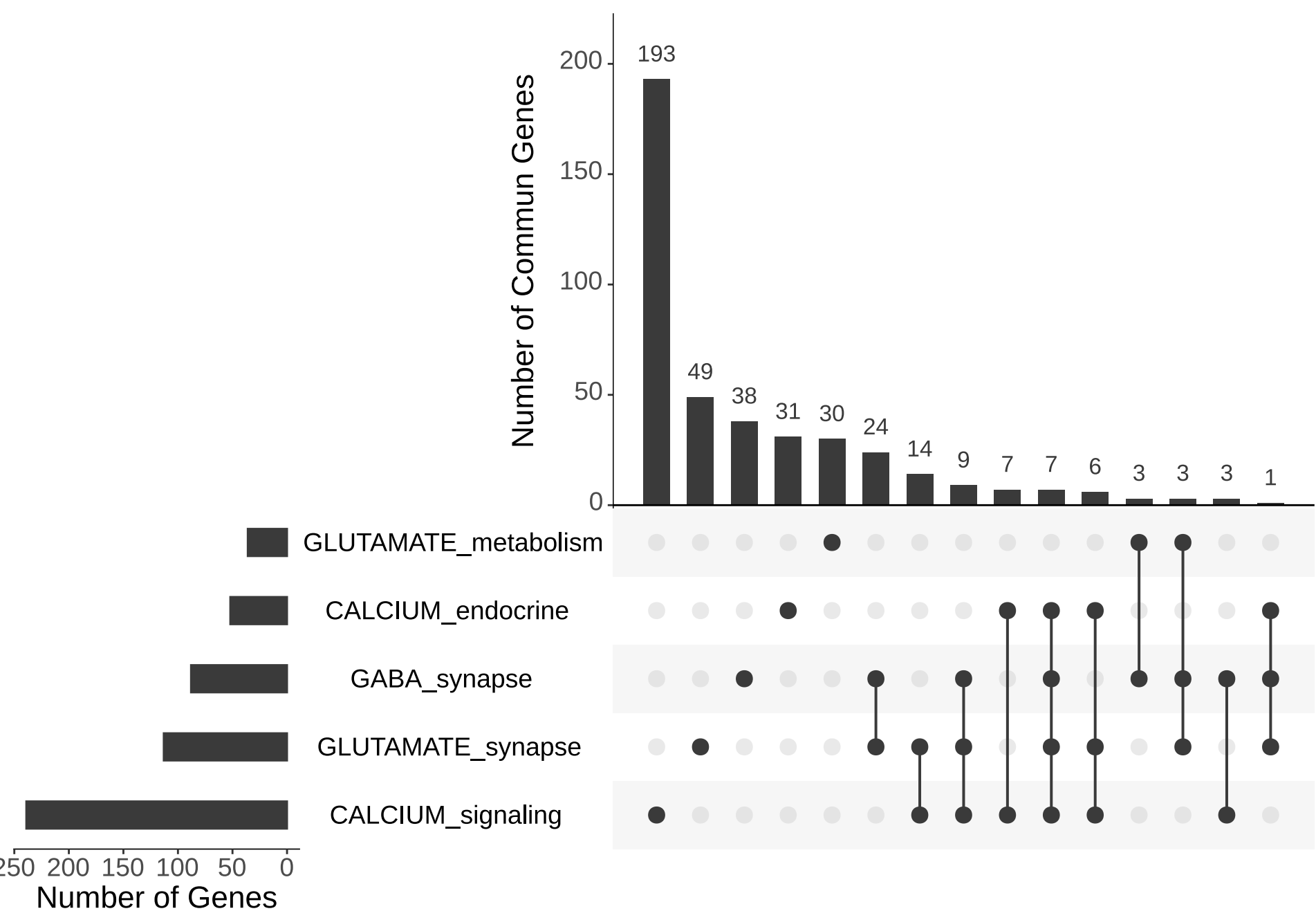

Supplement: Supplementary file 1 [file jpm-12-00633-s001.zip › supplementary_data_figureS1_upsetplot_neurotransmission_genes.pdf]

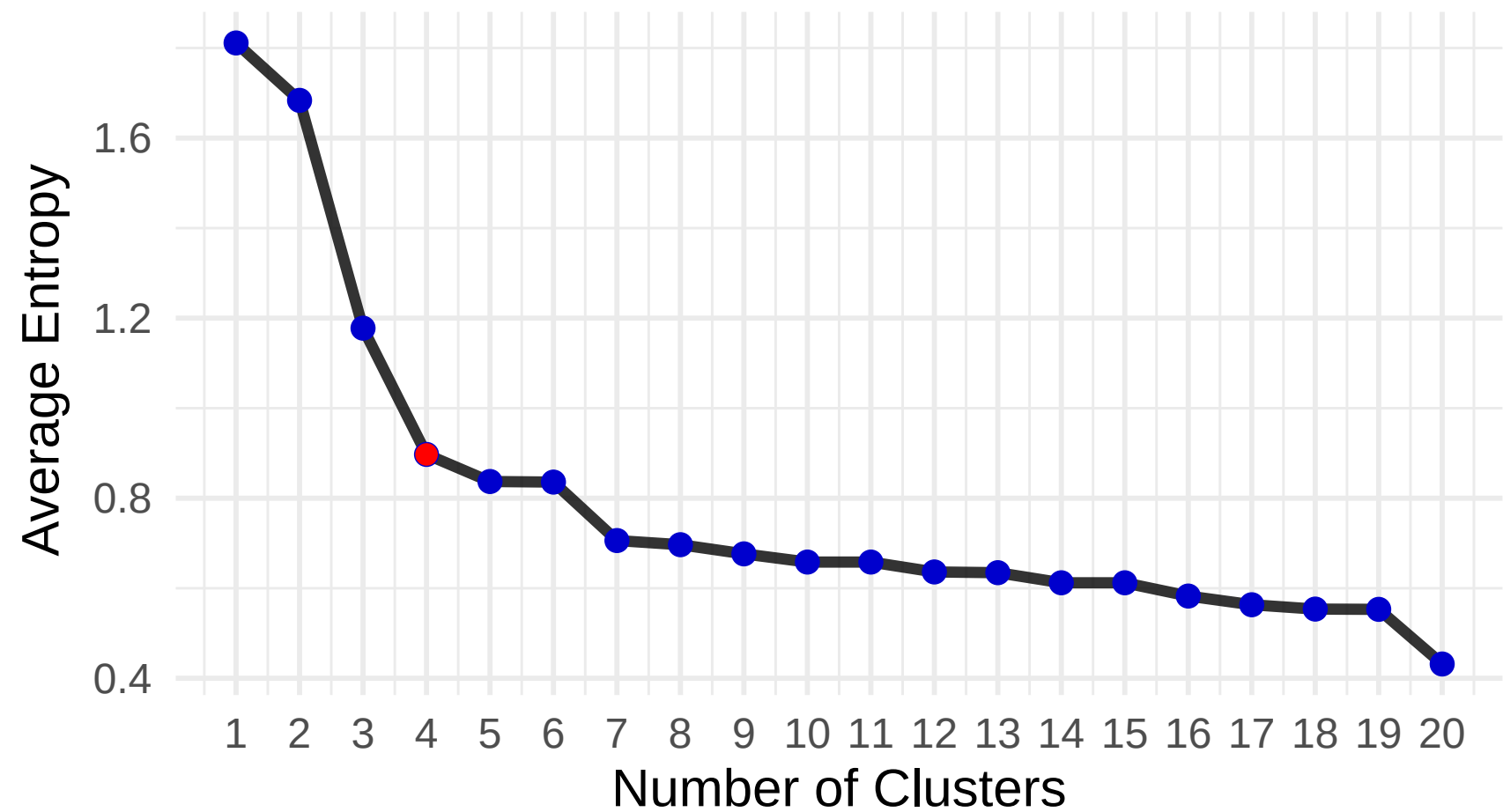

Supplement: Supplementary file 1 [file jpm-12-00633-s001.zip › supplementary_data_figureS2_entropy_curve.pdf]

Negative correlated-gene GO terms

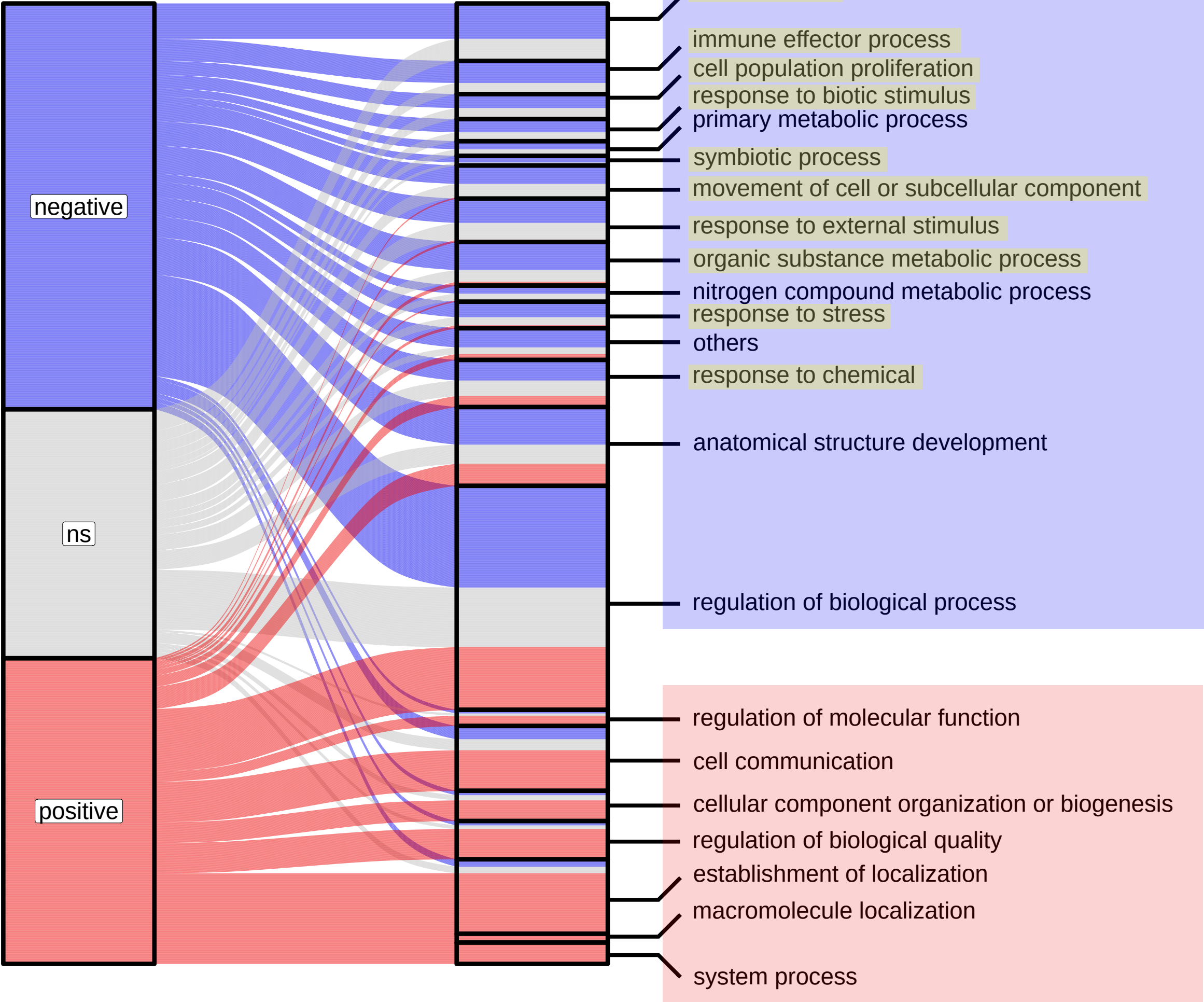

Supplement: Supplementary file 1 [file jpm-12-00633-s001.zip › supplementary_data_figureS3_correlations_sankeyplot_review.pdf]
